# Comparison of normalization methods for the analysis of metagenomic gene abundance data

**DOI:** 10.1186/s12864-018-4637-6

**Published:** 2018-04-20

**Authors:** Mariana Buongermino Pereira, Mikael Wallroth, Viktor Jonsson, Erik Kristiansson

**Affiliations:** Department of Mathematical Sciences, Chalmers University of Technology and University of Gothenburg, SE-412 96 Gothenburg, Sweden

**Keywords:** Shotgun metagenomics, Gene abundances, Normalization, High-dimensional data, Systematic variability, False discovery rate

## Abstract

**Background:**

In shotgun metagenomics, microbial communities are studied through direct sequencing of DNA without any prior cultivation. By comparing gene abundances estimated from the generated sequencing reads, functional differences between the communities can be identified. However, gene abundance data is affected by high levels of systematic variability, which can greatly reduce the statistical power and introduce false positives. Normalization, which is the process where systematic variability is identified and removed, is therefore a vital part of the data analysis. A wide range of normalization methods for high-dimensional count data has been proposed but their performance on the analysis of shotgun metagenomic data has not been evaluated.

**Results:**

Here, we present a systematic evaluation of nine normalization methods for gene abundance data. The methods were evaluated through resampling of three comprehensive datasets, creating a realistic setting that preserved the unique characteristics of metagenomic data. Performance was measured in terms of the methods ability to identify differentially abundant genes (DAGs), correctly calculate unbiased *p*-values and control the false discovery rate (FDR). Our results showed that the choice of normalization method has a large impact on the end results. When the DAGs were asymmetrically present between the experimental conditions, many normalization methods had a reduced true positive rate (TPR) and a high false positive rate (FPR). The methods trimmed mean of M-values (TMM) and relative log expression (RLE) had the overall highest performance and are therefore recommended for the analysis of gene abundance data. For larger sample sizes, CSS also showed satisfactory performance.

**Conclusions:**

This study emphasizes the importance of selecting a suitable normalization methods in the analysis of data from shotgun metagenomics. Our results also demonstrate that improper methods may result in unacceptably high levels of false positives, which in turn may lead to incorrect or obfuscated biological interpretation.

**Electronic supplementary material:**

The online version of this article (10.1186/s12864-018-4637-6) contains supplementary material, which is available to authorized users.

## Background

In shotgun metagenomics, microorganisms are studied by sequencing DNA fragments directly from samples without the need for cultivation of individual isolates [1]. Since shotgun metagenomics is culture-independent, it provides an efficient and unbiased way to describe microbial communities, their taxonomic structure and biochemical potential [2]. The increasing performance of high-throughput DNA sequencing technologies has rapidly expanded the potential of metagenomics, making it a key measurement technique in the analysis of the human microbiome and environmental microbial communities [3–6]. The data produced by shotgun metagenomics is often analyzed based on the presence of genes and their abundances in and between samples from different experimental conditions. The gene abundances are estimated by matching each generated sequence read against a comprehensive and annotated reference database [7–9]. The database typically consists of previously characterized microbial genomes, a catalog of genes or *de novo* assembled contiguous sequences. The gene abundances are then calculated by counting the number of reads matching each gene in the reference database. Finally, statistical analysis is used to identify the genes that have a significant differential abundance between the studied conditions.

Gene abundance data generated by shotgun metagenomics is however affected with multiple sources of variability which makes it notoriously hard to interpret [10–12]. A substantial part of this variability is systematic and affects multiple genes and/or samples in a similar way. One example of systematic variability is the differences in sequence depth, where each sample is represented by a varying number of reads [13]. Systematic variability also comes from other technical sources, such as inconsistencies in the DNA extraction and sample handling, varying quality between sequencing runs, errors in the read mapping, and incompleteness of the reference databases [14]. In addition, systematic variability may also be of biological nature, where, for example, sample-specific differences in average genome size, species richness and GC-content of the reads can affect the gene abundance [15, 16]. Regardless of its source, systematic variability significantly increases the variation between samples and thereby decrease the ability to identify genes that differ in abundance. Removal of systematic variability, a process referred to as normalization, is therefore vital to achieve a satisfactory statistical power and an acceptable FPR.

A wide range of different methods has been applied to normalize shotgun metagenomic data. The majority of these normalization methods are based on scaling, where a sample-specific factor is estimated and then used to correct the gene abundances. One approach is to derive the scaling factor from the total gene counts present in the sample [17, 18]. This enables removal of the often substantial differences in sequencing depth. However, the total gene counts is heavily dominated by the most abundant genes such that their variability may have a major impact on the scaling factor. To avoid the variability caused by high-abundant genes, the median and upper quartile normalization methods have been proposed as more robust alternatives [12, 19]. These methods estimate the scaling factors based on the 50th and 75th percentile of the gene count distribution, respectively. Similarly, the normalization method cumulative sum scaling (CSS) calculates the scaling factors as a sum of gene counts up to a threshold [20]. The method optimizes the threshold from the data in order to minimize the influence of variable high-abundant genes. Another method that robustly estimates the scaling factor is the TMM [21], which compares the gene abundances in the samples against a reference, typically set as one of the samples in the study. The scaling factor is then derived using a weighted trimmed mean over the differences of the log-transformed gene-count fold-change between the sample and the reference. Similarly to TMM, relative log expression (RLE) calculates scaling factors by comparing the samples to a reference [22]. However, in contrast to TMM, RLE uses a pseudo-reference calculated using the geometric mean of the gene-specific abundances over all samples in the study. The scaling factors are then calculated as the median of the gene counts ratios between the samples and the reference. A commonly used normalization method that is not based on scaling is rarefying, where reads in the different samples are randomly removed until the same predefined number has been reached, thereby assuring a uniform sequence depth [13, 23]. Another method that avoids scaling is the quantile-quantile normalization, in which the gene abundance distributions in different samples are made identical by adjusting their quantiles according to a reference distribution derived by averaging over all the samples [19, 24, 25].

Comparisons of normalization methods have previously been done for RNA-seq data [19, 26] as well as count data produced from the study of operational taxonomic units (OTUs) generated by amplicon sequencing [13, 23]. These studies found a large dependency between performance and data characteristics. Thus, it is likely that normalization methods that have previously been shown to perform well for other forms of count data are not appropriate for shotgun metagenomics. Indeed, metagenomic gene abundance data is almost always highly undersampled and plagued by high technical noise and biological between-sample variability, which makes it dependent on proper normalization [12]. However, no evaluation of data-driven normalization methods for shotgun metagenomics has been performed. It is therefore unclear how the normalization should be performed to ensure a correct interpretation of the end results.

To address this knowledge gap and to provide guidance in choosing a suitable data-driven normalization method, we have performed a systematic evaluation of nine methods on gene abundance data from shotgun metagenomics. The evaluation was performed on datasets formed by individual resampling of three comprehensive metagenomic datasets, thereby creating a realistic setting where the unique characteristics and variance structure of the data are preserved. The methods were evaluated based on their impact on the identification of DAGs by comparing their TPR and FPR as well as their ability to correctly estimate unbiased *p*-values and control the FDR. Our results showed that the normalization methods had a substantially different performance in identifying DAGs. Several of the methods demonstrated a high FPR, especially when the DAGs were distributed asymmetrically between the experimental conditions. In some cases, the high FPR also resulted in an unacceptably high FDR. TMM and RLE had the overall highest performance, with a high TPR, low FPR and a low FDR in most of the evaluated scenarios and are therefore recommended methods for normalization of gene abundance data. We conclude that the choice of normalization method is critical in shotgun metagenomics and may, if not done correctly, result in incorrect biological interpretations.

## Methods

### Normalization methods

In this study we evaluate the performance of nine normalization methods for count data, representing gene abundances from shotgun metagenomics (Table 1). Seven methods were scaling methods, where a sample-specific normalization factor is calculated and used to correct the counts, while two methods operate by replacing the non-normalized data with new normalized counts. Assume that gene abundance data is given as counts describing the number of DNA fragments sampled for each gene from a microbial community. Let *Y*_*ij*_ be the counts for gene *i*=1,…,*m* in sample *j*=1,…,*n*. The scaling normalization methods will derive a sample-specific normalization factor, denoted *N*_*j*_, while non-scaling methods will replace *Y*_*ij*_ with the normalized $\widetilde {Y}_{ij}$.

**Table 1:** Data-driven methods for normalization of shotgun metagenomic data included in this study

Method	Description	Availability
Total counts	Calculates scaling factors based on the total gene abundances	-
Median	Calculates scaling factors based on the median gene abundance	edgeR package in Bioconductor
Upper quartile [19]	Calculates scaling factors based on the upper quartile of the gene abundances	edgeR package in Bioconductor
Trimmed mean of *M*-values (TMM) [21]	Calculates scaling factors based on robust analysis of the difference in relative abundance between samples.	edgeR package in Bioconductor
Relative Log Expression (RLE) [30]	Calculates scaling factors using the ratio between gene abundances and their geometric mean	DESeq package in Bioconductor
Cumulative sum scaling (CSS) [20]	Calculates scaling factors as the cumulative sum of gene abundances up to a data-derived threshold	metagenomeSeq package in Bioconductor
Reversed cumulative sum scaling (RCSS)	Calculates scaling factors as the cumulative sum of high abundant genes	-
Quantile-quantile [19]	Transforms each sample to follow a data-derived reference distribution	-
Rarefying [55]	Randomly removes gene fragments until the sequencing depth is equal in all samples	phyloseq package in Bioconductor

**Total count** derives the normalization factor *N*_*j*_ as the sum of all gene counts in a sample *j* [17, 18, 27], i.e.

$$ N_{j} = \sum_{i=1}^{m} Y_{ij}. $$

Total count thus adjust the abundance of each gene based on the total number of DNA fragments that are binned in the sample. Total count was implemented in R (version 3.2.1) [28] using the ’colSums’ function.

**Median** calculates the the normalization factor *N*_*j*_ as the median of genes counts that are non-zero in at least one sample, i.e.

$$N_{j} = \underset{i \in G^{*}}{\text{median}}\ Y_{ij}, \quad G^{*} = \left\{i: \sum_{j=1}^{n} Y_{ij} > 0\right\}. $$

Median normalization provides a robust alternative to total counts that is less influenced by most highly abundant genes. Median normalization was performed using the edgeR Bioconductor package (version 3.10.5) [29].

**Upper quartile** estimates the normalization factor *N*_*j*_ as the sample upper quartile (75th percentile) of genes counts that are non-zero in at least one sample [19], i.e.

$$ N_{j} = \underset{i\in G^{*}}{\mathrm{upper~ quartile}}\ Y_{ij}, \quad G^{*} = \left\{i: \sum_{j=1}^{n} Y_{ij} > 0\right\}. $$

In contrast to median, the upper quartiles of the gene abundance distribution are used to calculate the scaling factors which aims to further increase the robustness. Upper quartile normalization was done using the edgeR Bioconductor package (version 3.10.5) [29].

**TMM** calculates the normalization factor *N*_*j*_ using a robust statistics based on the assumption that most genes are not differentially abundant and should, in average, be equal between the samples [21]. First, a sample *r* is chosen as reference. For each sample *j*, genes are filtered based on their mean abundance and fold-change between the sample and the reference. An an adjustment $f_{j}^{(r)}$ is then calculated as the mean of the remaining log fold-changes weighted by the inverse of the variance. The normalization factor is then given by

$$ N_{j} = f_{j}^{(r)} \sum_{i = 1}^{m} Y_{ij}. $$

TMM normalization was performed using the edgeR Bioconductor package (version 3.10.5), which, by default, trims 30% of log fold-change and 5% of mean abundance [29].

**RLE** assumes most genes are non-DAGs and uses the relative gene abundances to calculate the normalization factor [22]. First, a reference is created for each gene *i* by taking the geometric mean its abundances across all samples. The normalization factor *N*_*j*_ is then calculated as the median of all ratios between gene counts in sample *j* and the reference, i.e.

$$ N_{j} = \underset{i}{\text{median}}\frac{Y_{ij}}{\left(\prod_{j^{\prime} = 1}^{n} Y_{ij^{\prime}} \right)^{1/n}}. $$

Normalization using RLE was done using the DESeq2 Bioconductor package (version 1.14.1) [30].

**CSS** is based on the assumption that the count distributions in each sample are equivalent for low abundant genes up to a certain threshold $q_{j}^{\hat {l}}$, which is calculated from the data [20]. First, the median of each *l*th quantile across all samples is calculated. The threshold $q_{j}^{\hat {l}}$ is set as the largest quantile where the difference between the sample-specific quantiles is sufficiently small (measured based on the distance to the median quantile). Note that the threshold is set to be at least the 50th percentile. The normalization factor for sample *j* is then computed as the sum over the genes counts up to the threshold $q_{j}^{\hat {l}}$, i.e.

$$ N_{j} = \sum_{i:Y_{ij} \leq q_{j}^{\hat{l}}} Y_{ij}. $$

CSS normalization was done using metagenomeSeq Bioconductor package (version 1.10.0) [20].

**Reversed cumulative sum scaling** (RCSS) is a variant of CSS that utilize the observation that high-abundant genes in shotgun metagenomic data have, in general, a lower coefficient of variation [11]. RCSS therefore calculates the normalization factor *N*_*j*_ as the sum of all genes with an abundance larger than the median. The normalization factor is thus given by,

$$ N_{j} = \sum_{i:Y_{ij} \geq 0.5} Y_{ij}. $$

RCSS was implemented in R (version 3.2.1) [28] using the ‘colQuantiles’ function from ‘matrixStats’ package (version 0.51.0) and ’sum’ over a logical vector.

**Quantile-quantile** normalizes the data by transforming each sample to follow a reference distribution [19]. The reference distribution is calculated by taking the median of all quantiles across the samples, i.e.

$$ \bar{q}_{l} = \underset{j \in S}{\mathrm{~median~}} q^{l}_{j}, $$

where $q^{l}_{j}$ is the *l*th quantile in the *j*th sample. The counts *Y*_*ij*_ are then replaced by $\widetilde {Y}_{ij}$ such that $q^{l}_{j} = \bar q_{l}$. If two genes have same number of counts, i.e. *Y*_*aj*_=*Y*_*bj*_ for any *a*,*b*, such that *a*≠*b*, the choice of which gene receives which quantile is made randomly. We implemented quantile-quantile in R (version 3.2.1) [28] adapted from the algorithm presented in [24]. In order to preserve the discrete structure of the data, the median over the quantiles was calculated as outlined above and if the number of samples were even, one of the two middle values was randomly selected.

**Rarefying** is a normalization method that discards fragments from each sample until a predefined number of fragments is the same for all samples [13, 23]. For each sample, fragments are sampled without replacement. The fragments that are not selected in this process are discarded. In this study, the predefined number of fragments was set to the lowest sample size among all included in the dataset.

### Identification of differentially abundant genes

The number of counts *Y*_*ij*_ in gene *i* and sample *j* was modeled using a over-dispersed Poisson generalized linear model (OGLM) [31, 32], i.e.

$$ \log\left(\mathbb{E}\left[Y_{ij}|x_{j}\right]\right) = \alpha_{i} + \beta_{i}x_{j} + \log\left(N_{j}\right), $$

where, *α*_*i*_ is the log of the baseline counts expected for a gene *i*, *β*_*i*_ is the effect parameter that describes the relative abundance of gene *i* between the two conditions, and *x*_*j*_ is an indicator function, such that *x*_*j*_=1 if sample *j* belongs to condition 1 and 0, otherwise. The counts *Y*_*ij*_ is assumed to follow a Poisson distribution with a gene-specific scaling of the variance (i.e. the so called quasi-Poisson model). Furthermore, *N*_*j*_ was set to the factor corresponding to the method used to normalize the data. For method that does not use a normalization factor (rarefying and quantile-quantile normalization), *N*_*j*_ was set to 1. The model parameters *α*_*i*_ and *β*_*i*_ were estimated using maximum likelihood. Then, a gene is classified as a DAG using an *F*-test, which decides whether the model with an effect parameter is a better fit than the model without. FDR was estimated using the Benjamini-Hochberg algorithm [33]. The OGLM was chosen for identification of DAGs since it incorporates gene-specific between-sample variability and has previously been shown to have a high and robust performance for many forms of shotgun metagenomic data. For a comparison between statistical methods for identification of DAGs we refer the reader to [32].

### Datasets

The normalization methods had their performance evaluated in three different publicly available metagenomic datasets, here denoted Human gut I, Human gut II and Marine. Human gut I contained 74 samples of sequenced DNA from gut microbiome of control patients in a type-2 diabetes study [5]. The choice of only using the controls was done to exclude potentially large effects that may be present between the healthy and sick individuals in this study. The DNA was obtained from fecal samples, and it was sequenced using Illumina sequencing to an average of 3.2·10^7^ high quality reads per sample. Reads were mapped to a common gene catalog and quantified. The gene catalog was in turn mapped to eggNOG database v3.0 [34]. Human gut II contains 110 samples of sequenced DNA from microbiomes in the human gut of healthy individuals in North and South America [35]. DNA was sequenced using massively parallel sequencing (454 sequencing) with an average of 1.6·10^5^ reads per sample. The reads were downloaded from MG-RAST database [36], and translated into all six reading frames, which were in turn mapped to eggNOG database v4.5 [37] using HMMER [38]. Mapped reads with e-value of max 10^−5^ were kept. The Marine dataset contains a set of samples from TARA ocean project, a large oceanic metagenome study with a total of 243 samples collected in 68 different locations across the globe [6]. DNA was sequenced using Illumina sequencing resulting in an average of 3.2·10^8^ reads per sample. Reads were mapped to an oceanic gene catalog using MOCAT v1.2 [39] using the eggNOG database v3.0. The count data was received directly from the project authors. We selected the largest homogeneous experimental condition consisting of 45 metagenomes extracted from surface aquatic ocean samples using a filter sizes between.22 to 3 *μ*m. For all datasets, genes with more than 75% zeros or mean abundance less than three were excluded from the analysis, resulting in 3573, 2345 and 4372 genes for Human gut I, Human gut II and Marine, respectively. The count data used in this study is available at [40].

### Resampling of data

The normalization methods were evaluated on artificial data created by randomly sampling metagenomes without replacement from each of the comprehensive datasets. The artificial data was divided in two groups, representing two experimental conditions, each consisting of *m* samples. Differentially abundant genes were introduced by random selection of genes that had their number of observed DNA fragments in one of the groups downsampled. Thus, for gene *i* and sample *j*, the counts *Y*_*ij*_ were replaced with a number generated by sampling from a binomial distribution, such that

$$ \hat Y_{ij}|Y_{ij} \sim \text{Binomial}(Y_{ij}, q), $$

where *q* is the effect size describing the average fold-change in abundance. In the evaluation, the number of samples in the groups as well as the total number of DAGs, the distribution of DAGs between the groups and the effect size *q* were varied.

### Performance measures

The performance of the normalization methods was evaluated based on the TPR, which represents the ability to correctly identify the DAGs, and on the FPR, which represents the amount of non-DAGs that were incorrectly identified as DAGs. Given a ranking list of genes sorted based on their *p*-values calculated by the statistical analysis described above, the TPR and FPR at position *k* were calculated as

$$ TPR(k) = \frac{TP(k)}{\#\{DAGs\}} \text{~~ and} \quad FPR(k) = \frac{FP(k)}{\#\{\text{non-}DAGs\}}, $$

where *TP*(*k*) is the number of true positive above position *k*, *FP*(*k*) is the number of false positives above position *k* and *#*{*DAGs*} and *#*{non-*DAGs*} were the total number of DAGs and non-DAGs in the dataset, respectively. The true FDR (tFDR) at position *k* was calculated as

$$ tFDR(k) = \frac{FP(k)}{TP(k) + FP(k)}\text{.} $$

while the estimated FDR (eFDR) was given by the Benjamini-Hochberg algorithm [33]. All performance measures were calculated based on 100 resampled datasets. The cut-off position *k* was chosen as follows: for the TPR analysis *k* corresponds to the position where FPR is 0.01, for the FPR analysis *k* corresponds to the position where TPR is 0.50, and for tFDR analysis *k* is the position where eFDR is 0.05.

## Results

In this study, we compared the performance of nine normalization methods for shotgun metagenomic gene abundance data. The comparison was made on artificial data consisting of two groups, created by individual resampling without replacement of three comprehensive metagenomic datasets. In the resampling, DAGs were introduced by randomly selecting genes to have their number of counts in one of the two groups downsampled. Each artificial dataset was normalized using the nine different methods and the ability to correctly identify the DAGs was assessed. This set-up was used to investigate how the performance of the normalization methods changed under different characteristics of the data such as group size, proportion of DAGs and their distribution between the two groups.

**Fig. 1:**
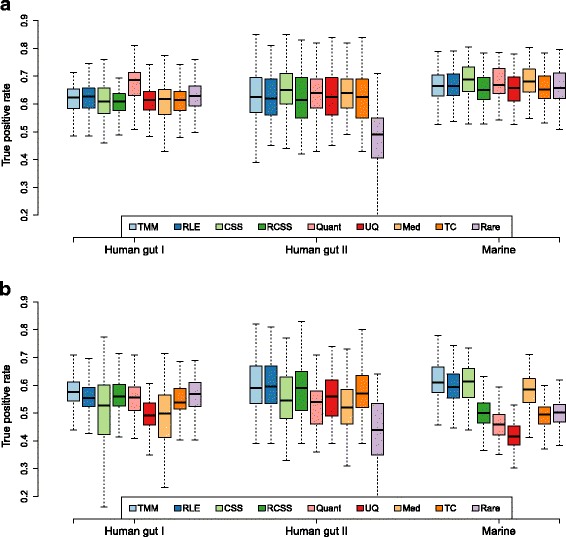
True positive rate analysis for group size 10+10. True positive rate at a fixed false positive rate of 0.01 (*y*-axis) for nine normalization methods and three metagenomic datasets (x-axis). The results were based on resampled data consisting of two groups with 10 samples in each, 10% DAGs with an average fold-change of 3. The DAGs were added in (**a**) equal proportion between the groups (‘balanced’) and in (**b**) in only one of the groups (’unbalanced’). The following methods are included in the figure: trimmed mean of *M*-values (TMM), relative log expression (RLE), cumulative sum scaling (CSS), reversed cumulative sum scaling (RCSS), quantile-quantile (Quant), upper quartile (UQ), median (Med), total count (TC) and rarefying (Rare)

First, all methods were evaluated with the DAGs symmetrically distributed between the two groups. Here, 10% of the genes were selected to be DAGs with an average fold-change of 3 and the group size was set to 10+10. The Human gut I and Marine datasets showed the overall highest performance for detecting DAGs (average TPR of all methods 0.63 and 0.67 respectively), while Human gut II, which had a substantially lower sequencing depth, had an average TPR of 0.61 (at a fixed FPR of 0.01, Fig. 1a and Table 2). Within each datasets, the normalization methods showed a similar performance. One exception was quantile-quantile that had a higher performance in Human gut I, with median TPR of 0.69 compared to the other methods that had a TPR around 0.62. Another exception was normalization using rarefying, which had a lower performance in Human gut II with a median TPR of 0.49 compared to the other methods with TPR of at least 0.62. In addition, CSS and median had a slighter higher performance in the Marine datasets, with TPR of 0.69 and 0.68, respectively, while other methods had a TPR around 0.66.

**Table 2:** True positive rate analysis for group size 10+10

Method	Human gut I		Human gut II		Marine	
	B	U	B	U	B	U
TMM	0.62	0.58	0.63	0.59	0.66	0.61
RLE	0.63	0.55	0.62	0.60	0.66	0.59
CSS	0.61	0.53	0.65	0.55	0.69	0.61
RCSS	0.61	0.56	0.62	0.59	0.65	0.50
Quantile-quantile	0.69	0.56	0.64	0.54	0.67	0.46
Upper quartile	0.61	0.49	0.63	0.56	0.66	0.42
Median	0.62	0.50	0.64	0.52	0.68	0.59
Total count	0.61	0.54	0.63	0.57	0.65	0.49
Rarefying	0.63	0.57	0.49	0.44	0.66	0.50

True positive rate at a fixed false positive rate of 0.01 for nine normalization methods and three metagenomic datasets using a group size of 10+10 for 10% DAGs with an average fold-change of 3.

B: balanced, 50% of effects added to each group.

U: unbalanced, 100% effects added to one group only

When effects instead were added in an unbalanced way, i.e. 10% DAGs added to the same group (Fig. 1b and Table 2), the performance of all methods decreased substantially, reducing the TPR, in average, with 9.0 p.p. (for an extended discussion on unbalanced DAGs in metagenomics see [41]). In this setting, upper quartile showed a TPR of 0.42 for the Marine dataset, which was, compared to its TPR of 0.66 in the balanced case, a reduction of 24 p.p.. The TPR of quantile-quantile normalization was also reduced to a TPR of 0.54 and 0.46 in the Human gut II and Marine datasets respectively. Reduced performance was also observed for CSS and median in at least one dataset (Table 2). The decrease in performance was, on the other hand, not as large for TMM and RLE which had a TPR between 0.55 and 0.61 for the three dataset corresponding to an average reduction in TPR of 4.5 and 5.6 p.p., respectively.

**Fig. 2:**
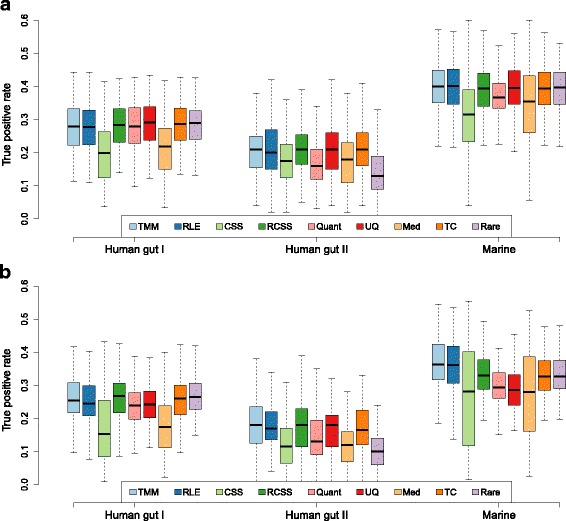
True positive rate analysis for group size 3+3. True positive rate at a fixed false positive rate of 0.01 (*y*-axis) for nine normalization methods and three metagenomic datasets (x-axis). The results were based on resampled data consisting of two groups with 3 samples in each, 10% DAGs with an average fold-change of 3. The DAGs were added in (**a**) equal proportion between the groups (‘balanced’) and in (**b**) only one of the groups (‘unbalanced’). The following methods are included in the figure: trimmed mean of M-values (TMM), relative log expression (RLE), cumulative sum scaling (CSS), reversed cumulative sum scaling (RCSS), quantile-quantile (Quant), upper quartile (UQ), median (Med), total count (TC) and rarefying (Rare)

Decreasing the group size to 3+3 resulted, as expected, in a reduced TPR (Fig. 2a and Table 3). For this group size, CSS and median had, compared to other methods, a particularly low performance. For example, in the balanced case in Human gut I, the TPR for these methods were 0.20 and 0.22 respectively, compared to other methods that had a TPR between 0.28 or 0.29. A similar trend was observed for quantile-quantile, which for larger group sizes was one of the highest performing methods. The trend of a substantially reduced TPR for CSS, median and quantile-quantile with reduced group size was further accentuated in the unbalanced case (Fig. 2b and Table 3). As previously, TMM and RLE had the overall highest TPR at low group sizes. Their performance was especially high in the Marine datasets, where the TPR was 0.36 for both TMM and RLE, respectively (Fig. 2b and Table 3).

**Table 3:** True positive rate analysis for group size 3+3

Methods	Human gut I		Human gut II		Marine	
	B	U	B	U	B	U
TMM	0.28	0.25	0.21	0.18	0.40	0.36
RLE	0.28	0.25	0.20	0.17	0.40	0.36
CSS	0.20	0.15	0.18	0.12	0.32	0.28
RCSS	0.28	0.27	0.21	0.18	0.39	0.33
Quantile-quantile	0.28	0.24	0.16	0.13	0.37	0.29
Upper quartile	0.29	0.24	0.21	0.18	0.40	0.29
Median	0.22	0.17	0.18	0.12	0.35	0.28
Total count	0.29	0.26	0.21	0.17	0.39	0.33
Rarefying	0.29	0.26	0.13	0.10	0.40	0.33

True positive rate at a fixed false positive rate of 0.01 for nine normalization methods and three metagenomic datasets using a group size of 3+3 for 10% DAGs with an average fold-change of 3.

B: balanced, 50% of effects added to each group.

U: unbalanced, 100% effects added to one group only

Next, we compared the results of the normalization methods with respect to the underlying gene abundance distributions. As expected, all scaling methods estimated scaling factors that were highly correlated with the average gene abundance (Additional file 1: Figure S1). Several of the methods estimated scaling factors that were highly correlated. The correlations were especially high between total counts and upper quartile (0.99), total counts and RCSS (0.99), upper quartile and RCSS (0.97) as well as TMM and RLE (0.952) (Additional file 2: Figure S2) suggesting that these methods are likely to generate similar normalization results. In contrast, the lowest correlations were found between CSS and RCSS (0.53), total counts and CSS (0.63) and median and RCSS (0.65). Furthermore, improper normalization is known to introduce false correlation between genes and to investigate this, we calculated the average pair-wise gene correlation before and after normalization (Additional file 3: Figure S3). Most normalization methods introduced a small increase in the gene-gene correlation. The increase was highest for upper quartile (0.035), total counts (0.027) and RCSS (0.027). However, no increase could be found for quantile-quantile and median.

**Fig. 3:**
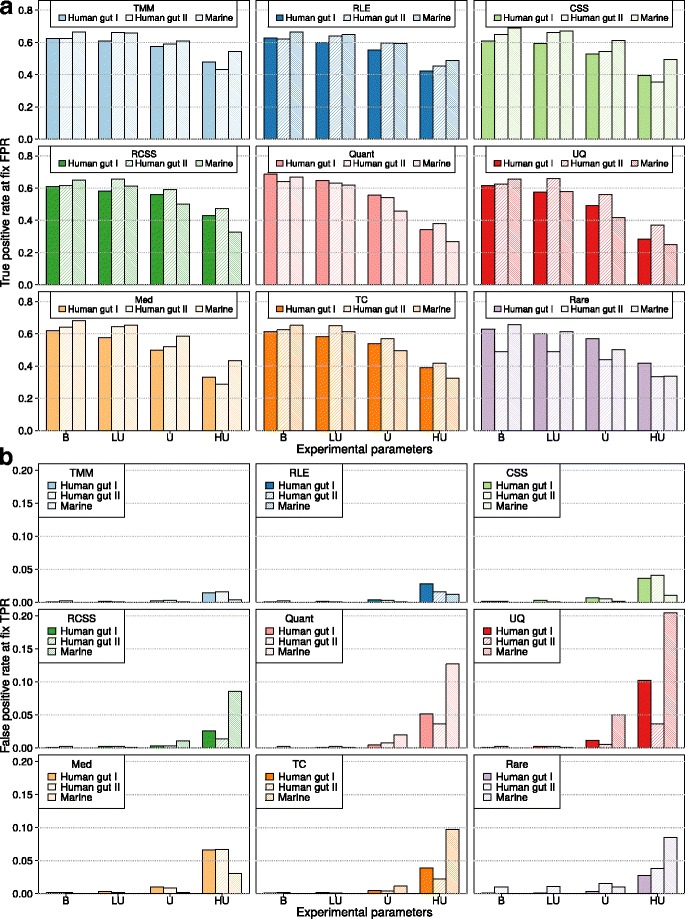
True and false positive rates for increasing unbalanced effects. (**a**) True positive rate at a fixed false positive rate of 0.01 (*y*-axis) and (**b**) false positive rate at a fix true positive rate of 0.50 (y-axis) for different distributions of effects between groups: balanced (’B’) with 10% of effects divided equally between the two groups, lightly-unbalanced (‘LU’) with effects added 75%-25% in each group, unbalanced (’U’) with all effects added to only one group, and heavily-unbalanced (‘HU’) with 20% of effects added to only one group (x-axis). The results were based on resampled data consisting of two groups with 10 samples in each and an average fold-change of 3. Three metagenomic datasets were used Human gut I, Human gut II and Marine. The following methods are included in the figure: trimmed mean of *M*-values (TMM), relative log expression (RLE), cumulative sum scaling (CSS), reversed cumulative sum scaling (RCSS), quantile-quantile (Quant), upper quartile (UQ), median (Med), total count (TC) and rarefying (Rare)

In order to further investigate the impact of unbalanced distribution of DAGs between groups on the normalization performance, we fixed the group size to 10+10 and the fold-change to 3, and compared all the methods under four different cases, each representing an increasing asymmetry of the distribution of DAGs: balanced effect (10% DAGs equally distributed over the two groups), lightly-unbalanced effects (10% DAGs, 75% in one group, 25% in the other group), unbalanced effects (10% DAGs, 100% in one group) and heavily-unbalanced effects (20% DAGs, 100% in one group). First, the impact of unbalanced DAGs on the methods performance was measured in terms of TPR at a fixed FPR of 0.01 (Fig. 3a and Additional file 4: Table S1). For all methods, the TPR was reduced with a more unbalanced effect added, and all methods had their lowest TPR at the heavily-unbalanced case. The reduction in TPR was lowest for TMM and RLE. For instance, in the Human gut I, TMM had a TPR of 0.62, 0.61, 0.58 and 0.48, for balanced, lightly-unbalanced, unbalanced and heavily-unbalanced cases respectively. The corresponding number for RLE was 0.63, 0.60, 0.55 and 0.42, while quantile-quantile showed 0.69, 0.65, 0.56 and 0.34, upper quartile 0.61, 0.57, 0.49 and 0.28 and median 0.62, 0.58, 0.50 and 0.33.

Next, we investigated the FPR at a fixed TPR of 0.50 (Fig. 3b and Additional file 5: Table S2). The trend was monotone with a increasing number of false positives for more unbalanced effects. Most methods had a low number of FPR for the balanced and lightly-unbalanced cases. Exception was rarefying, which for Human gut II, had an FPR of 0.011 already in the balanced case, while the other methods had an FPR of no more than 0.0022. In the unbalanced case, all methods showed an increased FPR. The increase was especially large for quantile-quantile (FPR of 0.050 in Marine), upper quartile (FPR of 0.020 in the Marine dataset) and rarefying (FPR of 0.010 in Human gut II). For the heavily-unbalanced case, the FPR was further increased. The levels were especially high for RCSS, quantile-quantile, upper quartile, median, total count and rarefying (Additional file 5: Table S2). It should be noted that for the Marine, upper quartile reached an FPR above 0.20, indicating that the number of false positives surpassed the number of added DAGs (Fig. 3b). TMM, RLE and CSS, on the other hand, presented an overall stable performance. For the heavily-unbalanced base, TMM had an FPR between 0.0036 to 0.016, RLE between 0.012 and 0.028 and CSS between 0.011 and 0.041 for all three datasets. Note that, the performance of all methods, both in terms of increased TPR and decreased FPR, was further pronounced when the fold-change was increased to 5, i.e. *p*=1/5 (Additional file 4: Table S1 and Additional file 5: Table S2).

In addition, we examined the bias of the effect size estimated by the OGLM under balanced, lightly-unbalanced, unbalanced and heavily-unbalanced cases (Additional file 6: Figure S4). For the balanced case, all methods resulted in estimated effect sizes close to the true fold-change of 3. However, when the effects became unbalanced several methods underestimated the effect size. This underestimation was especially large for CSS, upper-quartile and median. In particular, in the heavily-unbalanced case, median underestimated the effect size with more than 20%. In contrast, the estimates were less unbiased for TMM, RLE, RCSS, total counts and rarefying.

**Fig. 4:**
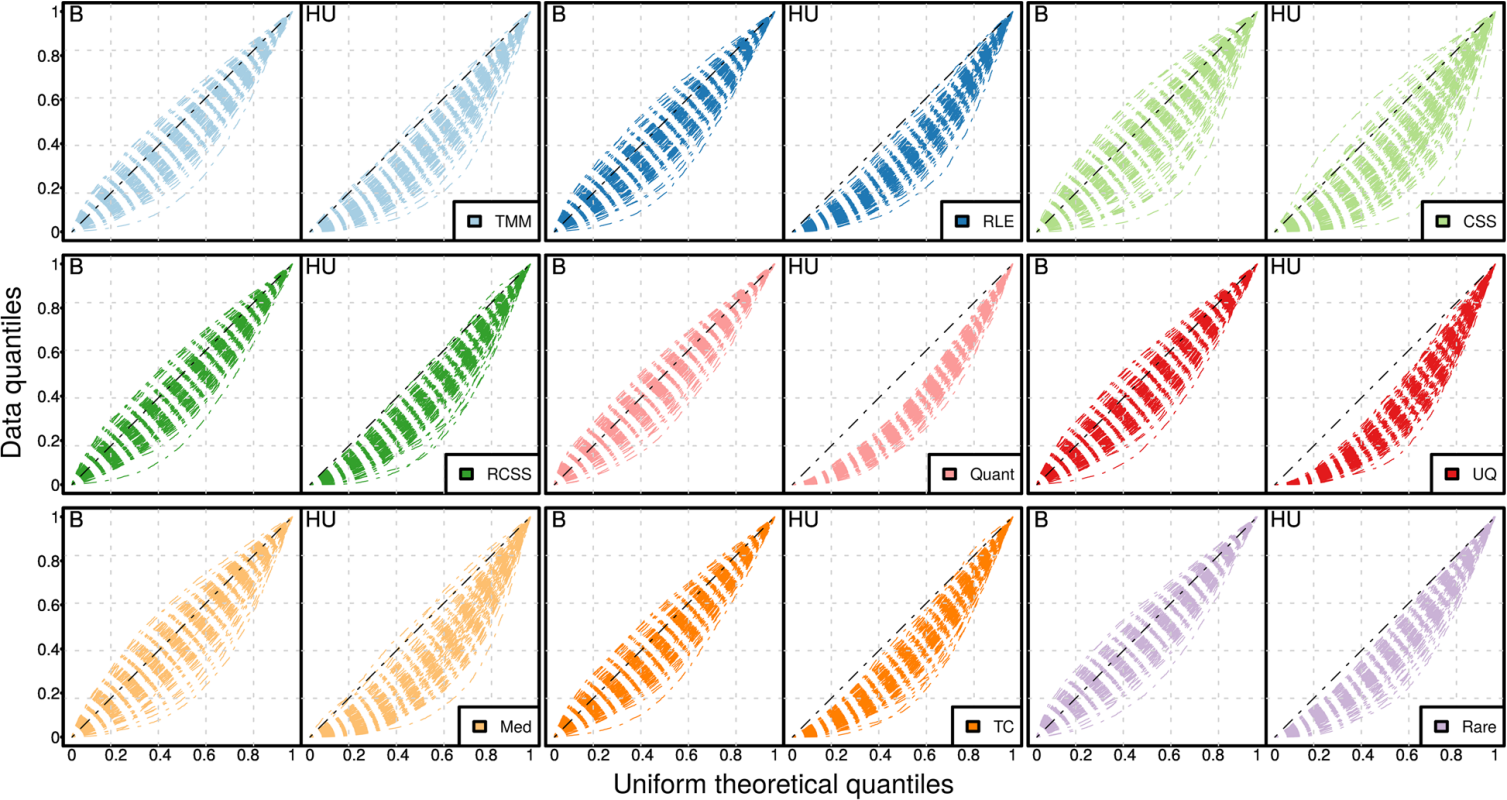
Quantile-quantile plots for *p*-values of non-DAGs. Data quantiles for the Human gut I dataset (*y*-axis) were plotted against the theoretical quantiles of the uniform distribution (x-axis) for nine normalization methods. The results were based on resampled data consisting of two groups with 10 samples in each, an average fold-change of 3, for balanced (‘B’) case where 10% effects were equally distributed in the two groups and heavily-unbalanced (‘HU’) case where 20% effects were added in only one group. Each dashed line is one of the 100 iterations. Lines deviating from the diagonal indicates biased *p*-values. The following methods are included in the figure: trimmed mean of M-values (TMM), relative log expression (RLE), cumulative sum scaling (CSS), reversed cumulative sum scaling (RCSS), quantile-quantile (Quant), upper quartile (UQ), median (Med), total count (TC) and rarefying (Rare)

False positives are often a result of a skewed non-uniform *p*-value distribution under the null hypothesis. We therefore examined the *p*-value distribution of the non-DAGs for the different normalization methods (Fig. 4). In the balanced case where the DAGs were symmetrically distributed over the groups, several methods, in particular TMM, quantile-quantile and total count, showed small but consistent trends towards too optimistic *p*-values, i.e. *p*-values that are smaller than expected compared to the uniform distribution. However, when the effect was changed to be heavily-unbalanced, the bias towards too optimistic *p*-values increased substantially for all methods. Methods producing the most biased *p*-value distributions were quantile-quantile and upper quartile. The bias was still present but not as serious for TMM, RLE, RCSS and CSS.

**Fig. 5:**
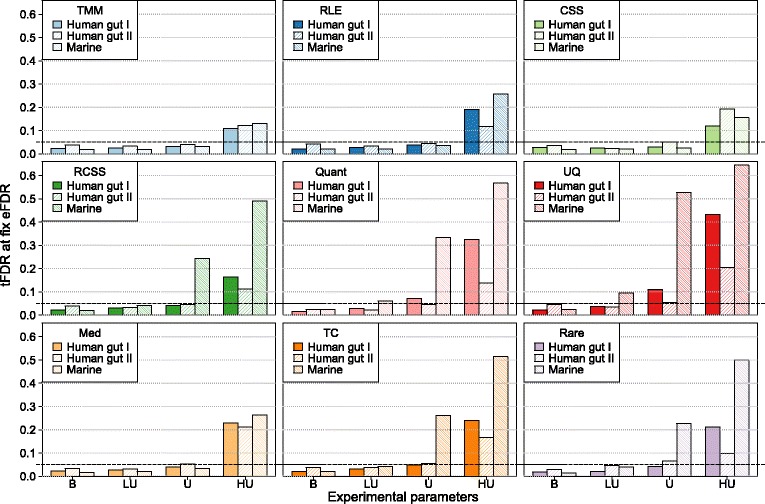
True false discovery rate at an estimated false discovery rate of 0.05 (*y*-axis) for different distribution of effects between groups (*x*-axis): balanced (‘B’) with 10% of effects divided equally between the two groups, lightly-unbalanced (‘LU’) with effects added 75–25% in each group, unbalanced (‘U’) with all effects added to only one group, and heavily-unbalanced (’HU’) with 20% of effects added to only one group. The results were based on resampled data consisting of two groups with 10 samples in each, and an average fold-change of 3. *P*-values where adjusted using Benjamini-Hochberg correction. Three metagenomic datasets were used Human gut I, Human gut II and Marine. The following methods are included in the figure trimmed mean of *M*-values (TMM), relative log expression (RLE), cumulative sum scaling (CSS), reversed cumulative sum scaling (RCSS), quantile-quantile (Quant), upper quartile (UQ), median (Med), total count (TC) and rarefying (Rare)

Finally, the ability to control the FDR was evaluated. For each normalization method, the true FDR was calculated at a fixed estimated FDR of 0.05 (Fig. 5 and Additional file 7: Tables S3). For the balanced case, all methods were conservative and showed a true FDR that was smaller than the estimated FDR. This changed, however, when the DAGs were added in an unbalanced way. For the Marine dataset in the lightly-unbalanced case, where 75% of the DAGs were added to one group, quantile-quantile and upper quartile showed a true FDR of 0.061 and 0.096, respectively, which was higher than the estimated 0.05. In the unbalanced case, five out of the nine methods were not able to control the FDR in at least one dataset. For instance, upper quartile demonstrated an especially large true FDR of 0.53 in the Marine. For heavily-unbalanced cases, none of the methods were able to control the FDR in any of the datasets. Still, TMM, RLE and CSS had a less biased true FDR than the other methods. In particular, the true FDR of TMM was close to 0.10 in all three datasets. On the other hand, RCSS, quantile-quantile, upper quartile, total count and rarefying resulted in unacceptably high FDRs (close to or above 50%) in at least one dataset.

## Discussion

In this paper, we compared nine methods for the normalization of metagenomic gene abundance data. The ultimate aim of the normalization step is the removal of unwanted systematic effects and thereby the reduction of the between-sample variability. This can significantly increase the ability to correctly identify DAGs, and to reduce the number of false positives. In this study, the normalization methods were therefore evaluated based on their statistical performance when identifying DAGs between experimental conditions. The performance was measured in terms of TPR, FPR, skewness of the *p*-value distributions and the ability to control the FDR. The comparison was done under realistic settings by utilizing artificial datasets created by individual resampling of three comprehensive metagenomic studies, representing both different forms of microbial communities and sequencing techniques. Our results showed that most of the included methods could satisfactory normalize metagenomic gene abundance data when the DAGs were equally distributed between the groups. However, when the distribution of DAGs become more unbalanced the performance was substantially reduced. In particular, many methods suffered from decreased TPRs, increased number of false positives and the inability to control the FDR. The size of the groups had also a major impact on the relative normalization results with several methods underperforming when only few samples were present.

TMM and RLE had the overall best performance, both in terms of TPR and FPR, for all three investigated datasets. Their performance, in relation to other methods, was especially high in the unbalanced case. In fact, TMM and RLE had an FPR less than 0.05 in all evaluations and datasets. TMM had, in most cases, slightly higher TPR and lower FPR than RLE, making it the highest performing method in this study. In addition, both TMM and RLE showed less biased estimates of the effect size and their estimated scaling factors showed high correlations. Larger effects on the gene abundances will significantly alter the count distributions which may result in incorrectly estimated scaling factors. TMM and RLE try to circumvent this problem by estimating the scaling factor from the relative difference of the gene abundance between the samples. For TMM, this procedure is done by comparison of samples against a reference sample and estimating scaling factors that minimize the pairwise differences. RLE estimates instead a reference by calculate the average gene abundance using a geometric mean. Scaling factors that minimize the difference between the each sample and reference are then calculated. By using robust statistics, both methods exclude genes that have a high relative difference, i.e. genes that are likely to be differentially abundant, which increase the accuracy of the estimated scaling factor. In contrast, the other scaling method included in this study (CSS, RCSS, upper quartile, median, and total count) estimates the scaling factors directly from the absolute gene abundances. This makes it harder to exclude differentially abundant genes and as a consequence, the scaling factors may become biased, especially when the effects are asymmetric. The high performance of TMM and RLE observed in this study is in line with previous evaluations on other forms of count data. For example, McMurdie et al. [13] showed that RLE had a high performance when normalizing data from operational taxonomic units (OTUs) generated by amplicon sequencing. Also, Dillies et al. [26] showed that TMM and RLE were the most efficient methods for reducing the between-sample variability in count data from RNA sequencing. Our results showed that this also holds true for shotgun metagenomic data and demonstrated that TMM and RLE increase the ability to identify DAGs and reduce the false positives. In addition to TMM and RLE, CSS showed a high overall performance for larger group sizes. CSS was particularly good at controlling the FDR, even when the effect was highly unbalanced. Even though CSS does not utilize the relative gene abundances, it tries to optimize what genes to include when calculating the scaling factor. This is done by summing low-abundant genes up to a cut-off that is adaptively selected from the data to minimize the variability. It should, however, be noted that CSS had among the worst performance for the low group size (Fig. 2), strongly suggesting that this method only should be applied to datasets with sufficiently many samples.

On the other end of the scale, normalization using quantile-quantile, median and upper quartile, as well as rarefying the data, had the overall lowest performance. The difference in TPR, compared to highest performing methods, was especially large in the heavily-unbalanced cases where DAGs were exclusively present in one of the samples. Taking the Marine dataset as an example, the TPR for these methods were 20 p.p. lower than TMM and RLE, which had the highest overall performance. All these methods also resulted in high FPRs that reached, in many cases, unacceptable levels. Among these low-performing methods, upper quartile and rarefying also resulted in inflated gene-gene correlation. Thus, quantile-quantile, median, upper quartile and rarefying are not recommended for normalization of metagenomic gene abundance data. Interestingly, the straight-forward total count method, which uses the total abundance of all genes in a sample as the basis for the normalization, had, overall, similar or higher performance than median and upper quartile. One argument for not using total count is that the sum of all gene abundances can be heavily dominated by the genes that are most commonly present in the microbial community. Instead, median and upper quartile should represent robust alternatives that avoid the most commonly present genes by replacing the sum with the 50th or 75th percentile of the gene count distribution as scaling factor. We did not, however, observe any tendencies that median or upper quartile had an overall higher performance than total count. On the contrary, the scaling factors estimated from total count and upper quartile had a very high correlation, which has also been shown in previous studies [42], suggesting that they produce a similar result. The performance of these methods were indeed similar with a small advantage for total count, which had an overall higher TPR and a lower FPR. It should, however, be pointed out that upper quartile has been developed specifically for transcriptomics, and has previously shown, using spike-in controls, to have a reasonable performance for normalization of RNA-seq [26]. Our results may therefore, at least partially, reflect differences in data structure between shotgun metagenomics and transcriptomics. It should, finally, be noted that the RAIDA R-package contains a normalization method for metagenomic gene abundances that has showed promising results for unbalanced effects [41]. However, this method is tightly connected with the specific log-normal statistical model implemented in the RAIDA package and since it is not generally applicable, there was no straightforward way to include it in this comparison.

The *p*-values for non-DAGs should, in theory, follow a uniform distribution. Our results showed that this was not the case. In the situations where the DAGs where distributed between the groups in a balanced way, all methods generated *p*-value distributions that were approximately uniform. However, when the DAGs were only present in one of the groups, the *p*-value distribution of the non-DAGs became skewed against low values. Metagenomic gene abundances are measured relatively to the sequencing depth and genes that are differentially abundant will therefore, indirectly, also affect non-DAGs. If a normalization method fails to compensate for this ’artificial’ effect, it may result in too low *p*-values for non-DAGs and, in turn, in an excessive number of false positives. Our results showed that quantile-quantile and upper quartile normalization methods had the most biased *p*-values, suggesting that this, at least partially, is likely the cause for their high FPR. Furthermore, previous studies have shown that statistical models for identification of DAGs in shotgun metagenomics can result in highly biased *p*-values if their underlying assumptions are invalid [32]. In particular, gene count models that does not incorporate gene-specific variability, such as the popular Fisher’s exact test, can incorrectly interpret high overdispersion as biological effects which may result in large numbers of false positives [11, 43]. It should be emphasized that in contrast to the biased *p*-values caused by invalid model assumptions, the skewed p-value distributions generated by improper normalization observed in this study, can not be addressed by replacing the parametric model (the overdispersed Poisson model), with e.g. a non-parametric method or a permutation-based approach.

The FDR is used to control the error rate in multiple testing of high-dimensional data [33]. Correct estimation of the FDR is highly dependent on a uniform *p*-value distribution for non-DAGs. Biased FDR estimation may result in a large number of false positives genes, i.e. non-DAGs incorrectly reported as significant. Our results showed that all normalization methods achieved a correctly estimated FDR when the effects were balanced. However, similarly to the *p*-values, the FDR became biased when the DAGs were introduced in an unbalanced way. Already at the lightly-unbalanced case, where effects were added to 10% of the genes distributed 75%-25% between the groups, two methods (quantile-quantile, upper quartile) were unable to control the FDR for at least one dataset. At the unbalanced case (10% of the genes set as DAGs, all in one group), six of the nine methods resulted in considerably biased FDR estimates. Only TMM, RLE and CSS were able to correctly control the FDR and only showed a moderate bias at the heavily-unbalanced case (20% of the genes set as DAGs, all in one group). Several of the other methods however showed an unacceptable FDR bias. In particular, RCSS, quantile-quantile, upper quartile and total count had a true FDR close to 50% when the corresponding estimated FDR was fixed to 5%. Our results thus show that many normalization methods produce highly skewed *p*-value distribution, which results in biased FDRs, as soon as the DAGs becomes unbalanced between the groups. It is worth to note that changing the approach for controlling the FDR to the more conservative Benjamini-Yekutieli method or the Storey q-values method did not remove the bias or resulted in a considerably reduced statistical power (Additional file 8: Figure S5 and Additional file 9: Figure S6, respectively). Controlling the number of false positive genes is vital in high-throughput data analysis [44, 45], since a high proportion of false positive can result in incorrect interpretation of the results and, in worst case, wrong biological conclusions. Using a normalization method that can reliably analyze gene abundance from shotgun metagenomics data without generating an unacceptably high false positive rate is thus vital for statistically sound results.

Rarefying normalizes count data by randomly removal of DNA fragments until all samples have the same predefined sequencing depth. Rarefying is commonly used in metagenomics [46–48] and has been both argued for and against in recent studies [13, 23]. In the present work, we showed that rarefying had a relatively low performance for normalization of metagenomic gene abundance data, both in terms of TPR, FPR and the ability to control the FDR. Since the ability to correctly identify DAGs increase with increasing number of DNA fragments, discarding data, as done by the rarefying method, has a negative effect on the performance. The performance was particularly low for the Human gut II dataset, where the TPR was low in all tested cases (e.g. Figs. 1a and 3a). Human gut II had the lowest sequencing depth of the datasets used in this study, and the effect was therefore most visible here. However, even for the two other datasets, which had more than 200-fold larger number of DNA fragments, rarefying still was among the methods with the lowest performance. For instance, in the Marine dataset (Fig. 5), rarefying resulted in a highly biased FDR estimation for both the unbalanced and the heavily-unbalanced cases. The low performance of rarefying in the datasets with high sequencing depth can, at least partially, be explained by the fact that genes that are low-abundant in a community, are also in general represented by few DNA fragments even in datasets with high sequencing depth, and discarding reads will have a particularly negative effect on these genes. Our results are thus in line with [13], who has previously demonstrated that rarefying has a low performance on count data from OTUs generated by amplicon sequencing. It should, in this context, be pointed out that there are situations where rarefying data may be necessary. This includes, for example, the estimation of diversity indices that are dependent on the sequencing depth and that have no straight-forward to incorporate a normalization scaling factor. However, for the identification of DAGs, the use of rarefying as a method to correct for differences in sequencing depth should be avoided.

The evaluation of normalization methods presented in this study was based on artificial gene count data generated by individual resampling three comprehensive metagenomic datasets. DAGs were introduced into the data by downsampling selected genes to simulate a lower abundance within the community. Thus, our setup was non-parametric and conserves important parts of the complex variance structure present in real metagenomic data. This includes, for example, the underlying discrete count distributions, the between-gene correlation and the sparsity of the data. In contrast, data used in previous studies (e.g. [13, 23, 26]) were simulated from parametric distributions and thus represent highly idealized cases. Even though our results are based on real metagenomic data, there are still specific assumptions made that are likely not to be true. The study is, for example, based on three datasets, which is too few to cover the full heterogeneity of the data generated within the field of metagenomics. Also, the resampling to form the artifical datasets was done independently which removes any correlations that may exist between the metagenomic samples. The downsampling used to create DAGs was done independently between the genes, disregarding correlations between effects which has previously been observed in microbial communities [49, 50]. Furthermore, some of the analyzed cases, in particular when all effects were added only to one experimental group, may be unrealistic and not common for many forms of metagenomic experiments. Nevertheless, unbalanced distribution of DAGs is not uncommon in metagenomic data, and may, for example, be a result of a strong selection pressures affecting one of the experimental groups [51–53]. Also, the nature of the effect is also often hard to predict a priori and normalization methods that do not have an overall high performance should therefore be avoided. The results from the current study should, ideally, be complemented with data that closer resemblance true metagenomic studies. However, a comprehensive reference dataset for shotgun metagenomics, similarly to SEQC in transcriptomics [54], needs to be established before such an analysis can be performed. Nevertheless, even if our data generation approach did not reflect all the nuances of metagenomic data and our evaluated cases did not represent all possible forms of biological effects, we argue that our approach is more sound and provides considerably more realistic results than method comparisons based on simulated data from parametric distributions.

## Conclusion

In conclusion, our evaluation showed that the choice of normalization method can greatly affect the quality of the results in the analysis of gene abundances in shotgun metagenomic data. When DAGs were asymmetrically distributed between experimental conditions, several well-established normalization methods showed a decreased TPR and an increased FPR. The high FPR resulted, for many methods, in an unacceptably biased FDR which can lead to a large number of false positives. The highest performing normalization methods in our study were TMM and RLE, and for larger group sizes CSS, which showed a high TPR and low FPR. These methods were also the best in controlling the FDR. Normalization is an essential step in the analysis of gene abundances in shotgun metagenomics. Our results emphasize the importance of selecting a sound and appropriate method for this task. They also demonstrates that the use of inappropriate normalization methods may obscure the biological interpretation of data. Further research for improved data-driven normalization of shotgun metagenomic data is therefore warranted.

## Additional files

Additional file 1 **Figure S1.** Histograms of Spearman correlations between normalization factors and raw counts of non-differentially abundant genes (non-DAGs). Spearman correlations were compute per gene in the Human gut I, for group size 10+10, with 10% of effects divided equally between the two group, and fold-change 3. Affected genes were randomly selected in 100 iterations. The following methods are included in the figure trimmed mean of M-values (TMM), relative log expression (RLE), cumulative sum scaling (CSS), reversed cumulative sum scaling (RCSS), upper quartile (UQ), median (Med) and total count (TC). (PDF 76 kb)

Additional file 2 **Figure S2.** Scatterplot of normalization factors for each pair of scaling methods. Normalization factors estimated per sample in the Human gut I, for group size 10+10, with 10% of effects divided equally between the two group, and fold-change 3. Affected genes were randomly selected in 100 iterations. The number on the top-left of each plot indicates the Spearman correlation for the normalization factors presented in the plot. The following methods are included in the figure trimmed mean of M-values (TMM), relative log expression (RLE), cumulative sum scaling (CSS), reversed cumulative sum scaling (RCSS), upper quartile (UQ), median (Med) and total count (TC). (PDF 316 kb)

Additional file 3 **Figure S3.** Mean Spearman correlation between raw and normalized counts. Spearman correlations were compute per gene before and after normalization in the Human gut I, for group size 10+10, with 10% of effects divided equally between the two group, and fold-change 3. Affected genes were randomly selected in 100 iterations. The following methods are included in the figure trimmed mean of M-values (TMM), relative log expression (RLE), cumulative sum scaling (CSS), reversed cumulative sum scaling (RCSS), quantile-quantile (Quant), upper quartile (UQ), median (Med), total count (TC) and rarefying (Rare). (PDF 8 kb)

Additional file 4 **Table S1.** True positive rate at a fixed false positive rate of 0.01 for a group size of 10+10. (PDF 16 kb)

Additional file 5 **Table S2.** False positive rate at a fix true positive rate of 0.50 for a group size of 10+10. (PDF 16 kb)

Additional file 6 **Figure S4.** Effect size analysis of DAGs. Estimated effect size of differentially abundant genes (DAGs) (*y*-axis) for different distribution of effects between groups (x-axis): balanced (‘B’) with 10% of effects divided equally between the two groups, lightly-unbalanced (’LU’) with effects added 75%-25% in each group, unbalanced (‘U’) with all effects added to only one group, and heavily-unbalanced (’HU’) with 20% of effects added to only one group (x-axis). The results were based on resampled data consisting of two groups with 10 samples in each, and an average fold-change of 3. Three metagenomic datasets were used Human gut I, Human gut II and Marine. The following methods are included in the figure trimmed mean of *M*-values (TMM), *relative log expression (RLE)*, cumulative sum scaling (CSS), reversed cumulative sum scaling (RCSS), quantile-quantile (Quant), upper quartile (UQ), median (Med), total count (TC) and rarefying (Rare). (PDF 436 kb)

Additional file 7 **Table S3.** True false discovery rate at an estimated false discovery rate of 0.05 for a group size of 10+10. (PDF 16 kb)

Additional file 8 **Figure S5.** True false discovery rate for *p*-values adjusted using Benjamini-Yekutieli method at an estimated false discovery rate of 0.05 (*y*-axis) for different distribution of effects between groups (*x*-axis): balanced (‘B’) with 10% of effects divided equally between the two groups, lightly-unbalanced (’LU’) with effects added 75%-25% in each group, unbalanced (‘U’) with all effects added to only one group, and heavily-unbalanced (’HU’) with 20% of effects added to only one group. The results were based on resampled data consisting of two groups with 10 samples in each, and an average fold-change of 3. Three metagenomic datasets were used Human gut I, Human gut II and Marine. The following methods are included in the figure trimmed mean of *M*-values (TMM), relative log expression (RLE), cumulative sum scaling (CSS), reversed cumulative sum scaling (RCSS), quantile-quantile (Quant), upper quartile (UQ), median (Med), total count (TC) and rarefying (Rare). (PDF 40 kb)

Additional file 9 **Figure S6.** True false discovery rate for *p*-values adjusted using Storey q-values method at an estimated false discovery rate of 0.05 (*y*-axis) for different distribution of effects between groups (*x*-axis): balanced (‘B’) with 10% of effects divided equally between the two groups, lightly-unbalanced (‘LU’) with effects added 75–25% in each group, unbalanced (‘U’) with all effects added to only one group, and heavily-unbalanced (‘HU’) with 20% of effects added to only one group. The results were based on resampled data consisting of two groups with 10 samples in each, and an average fold-change of 3. Three metagenomic datasets were used Human gut I, Human gut II and Marine. The following methods are included in the figure trimmed mean of M-values (TMM), relative log expression (RLE), cumulative sum scaling (CSS), reversed cumulative sum scaling (RCSS), quantile-quantile (Quant), upper quartile (UQ), median (Med), total count (TC) and rarefying (Rare). (PDF 132 kb)

## Abbreviations

B: Balanced
CSS: Cumulative sum scaling
DAG: Differentially abundant gene
eFDR: Estimated false discovery rate
FDR: False discovery rate
FPR: False positive rate
HU: Heavily-unbalanced
LU: Lightly-unbalanced
Med: Median
OGLM: Over-dispersed Poisson generalized linear model
OTU: operational taxonomic unit
Quant: Quantile-quantile
Rare: Rarefying
RCSS: Reversed cumulative sum scaling
RLE: Relative log expression
TC: Total count
tFDR: True false discovery rate
TMM: Trimmed mean of *M*-values
TPR: True positive rate
U: Unbalanced
UQ: Upper quartile
