# Supplementary figures and images for: Comparison of normalization methods for the analysis of metagenomic gene abundance data
Source: BMC Genomics. 2018 Apr 20;19:274. doi: 10.1186/s12864-018-4637-6 (PMC5910605; doi:10.1186/s12864-018-4637-6)

**TMM**

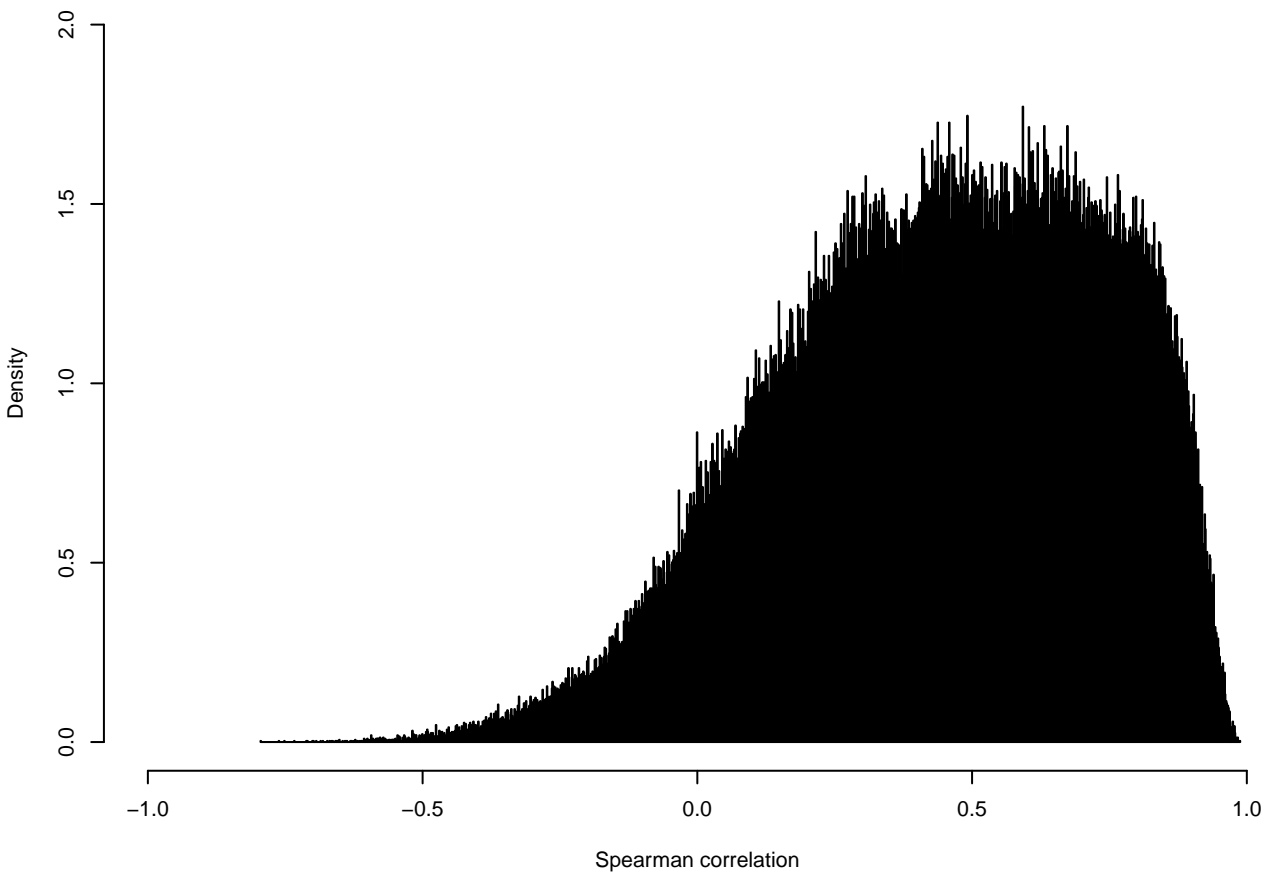

**RLE**

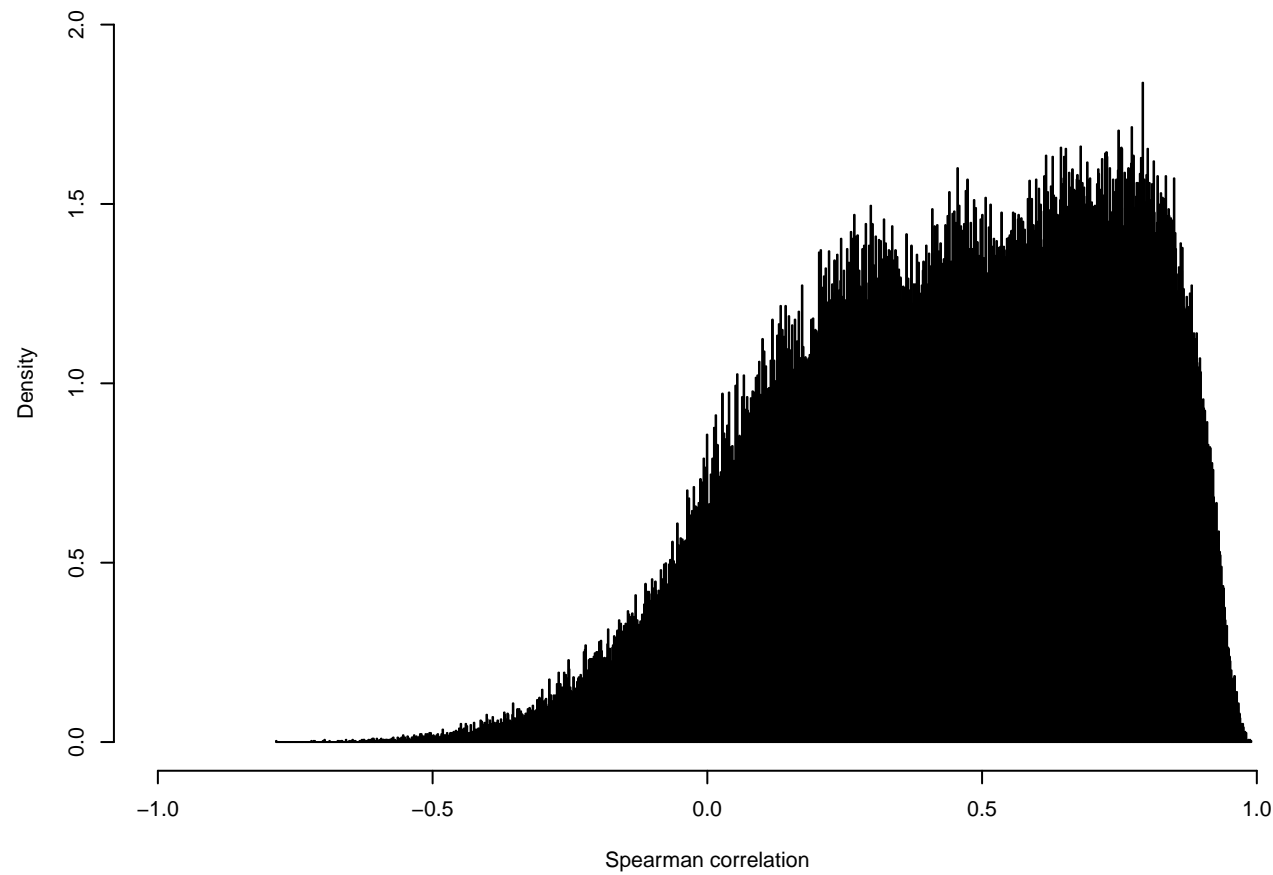

**CSS**

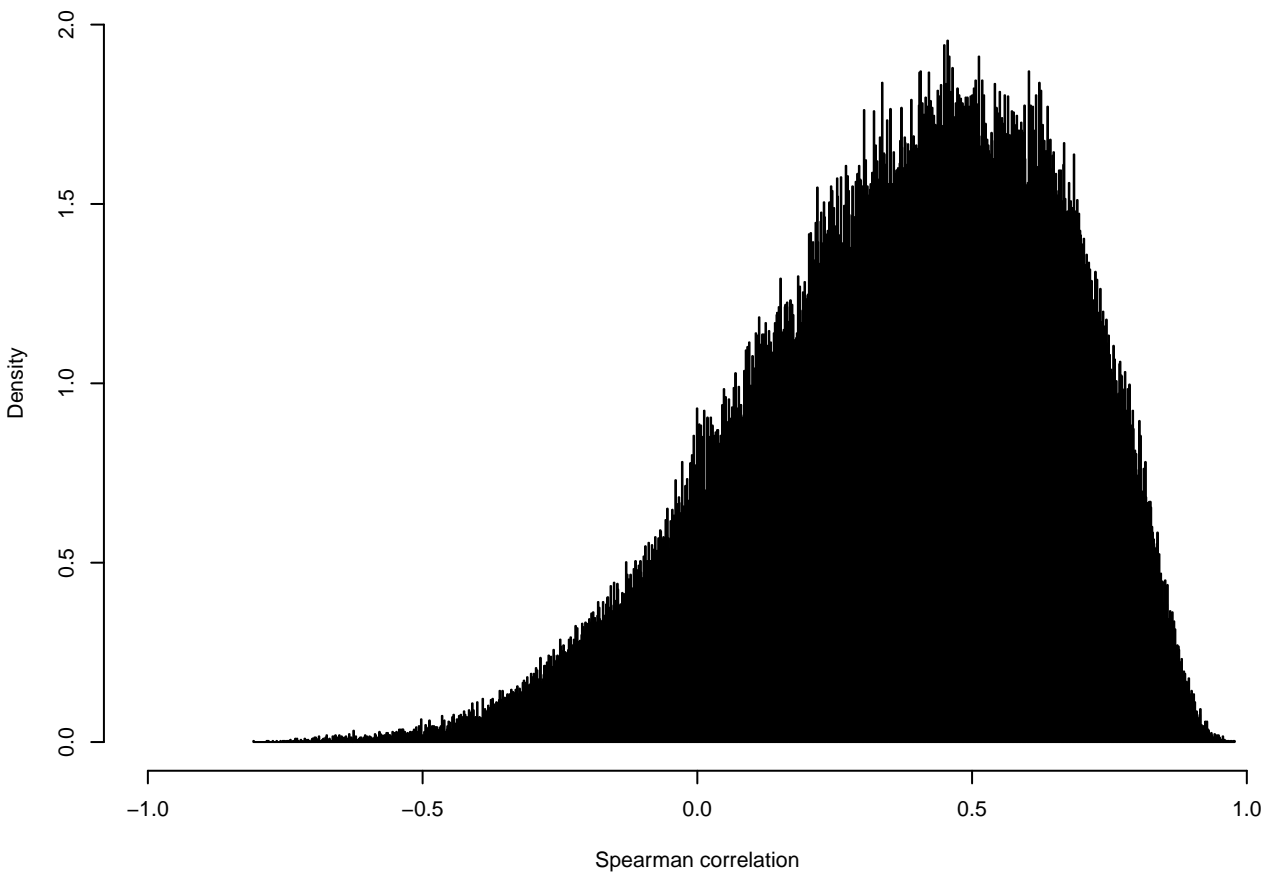

**RCSS**

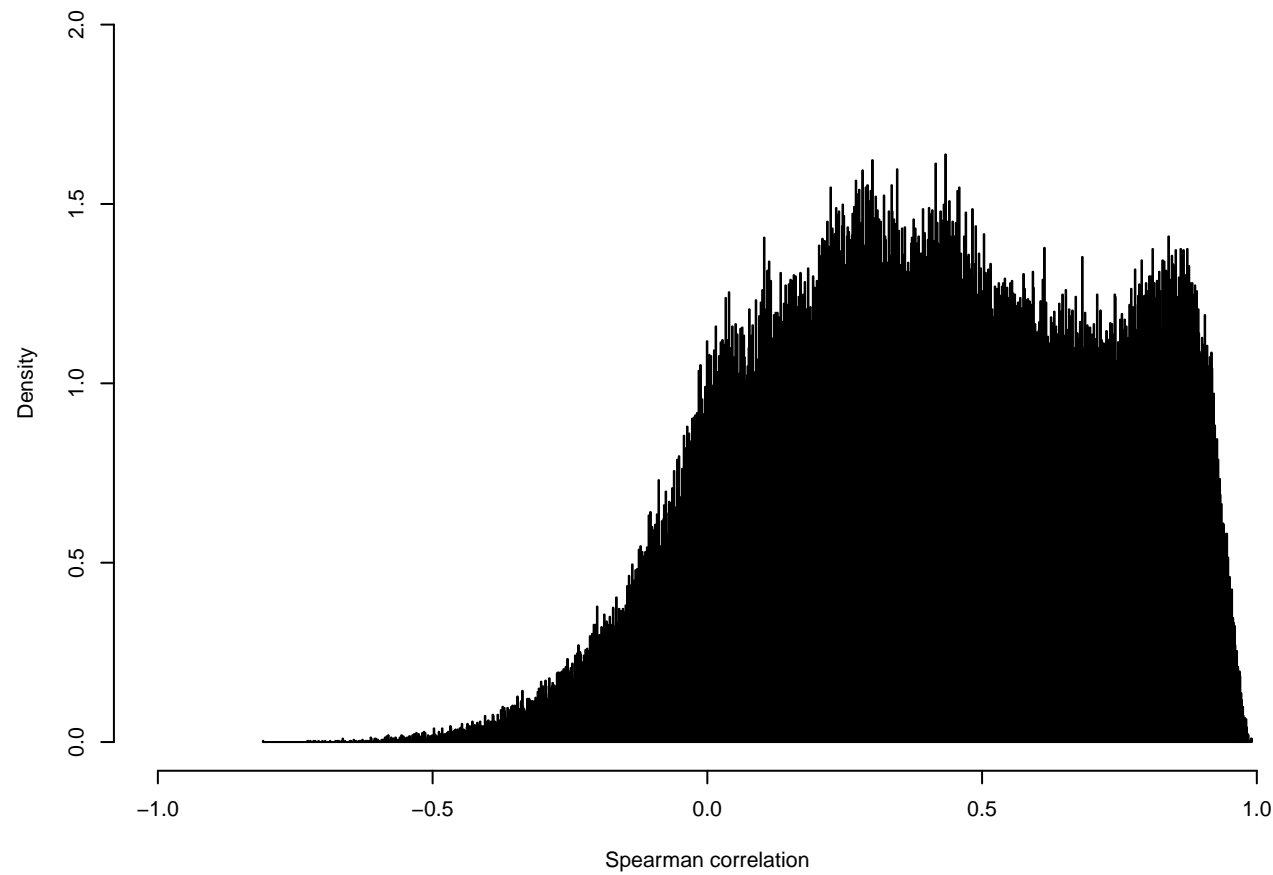

**UQ**

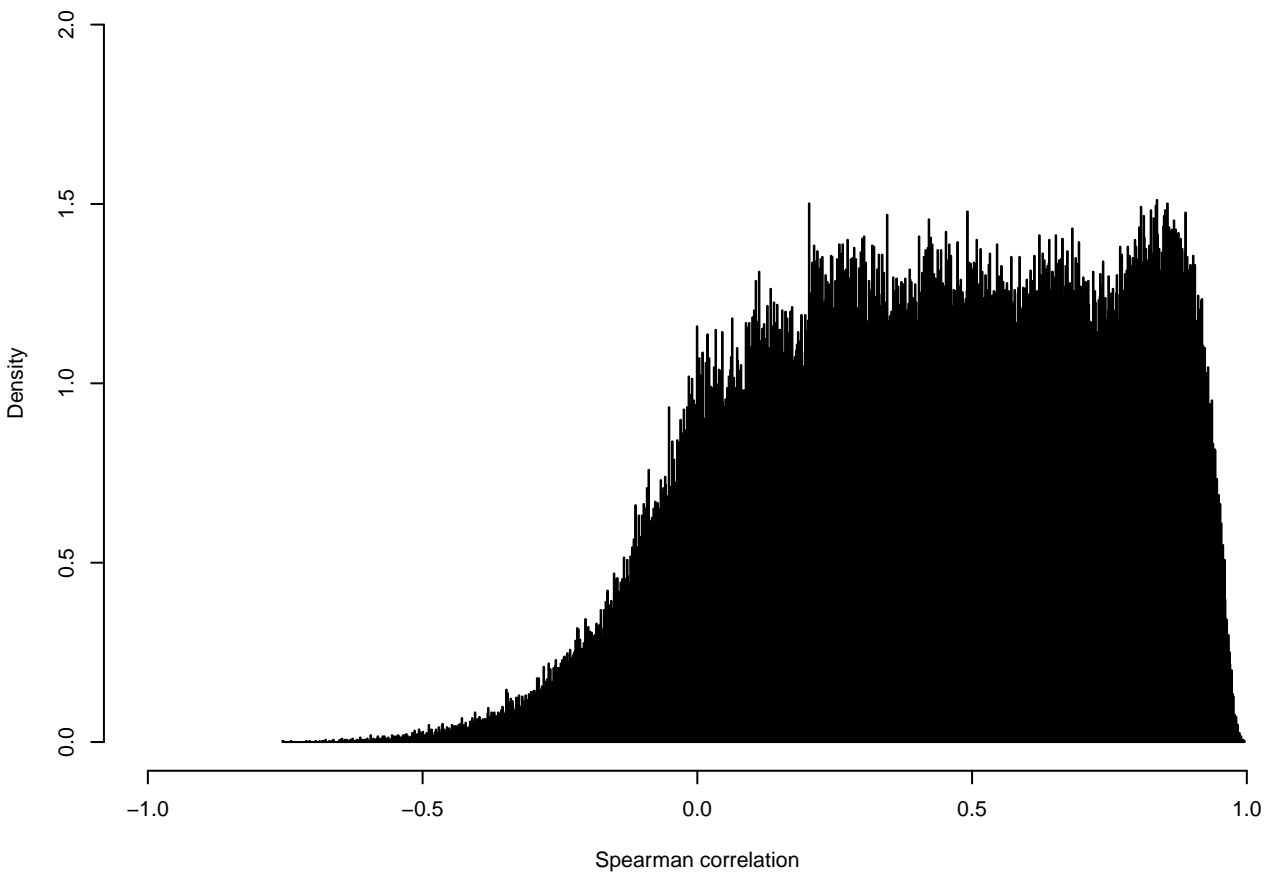

**Med**

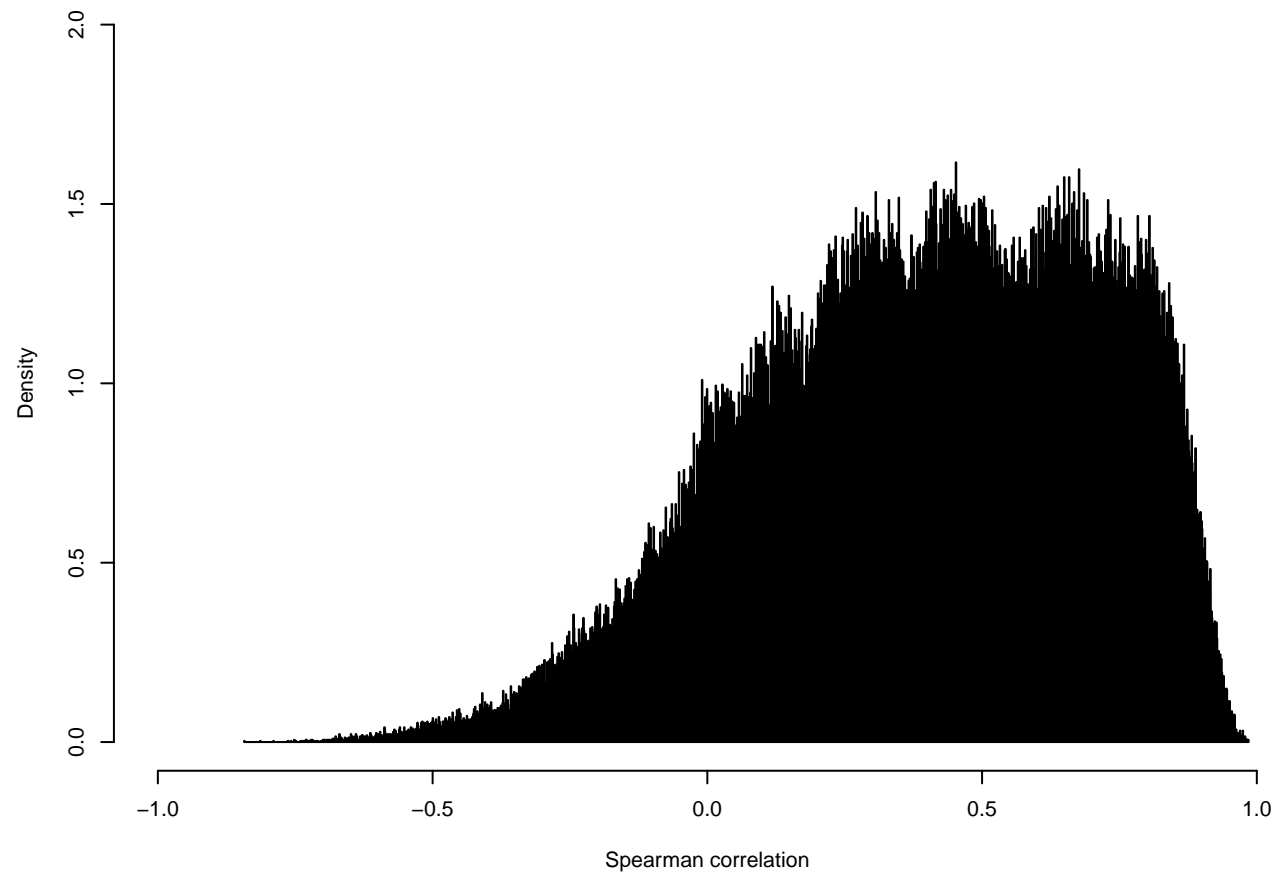

**TC**

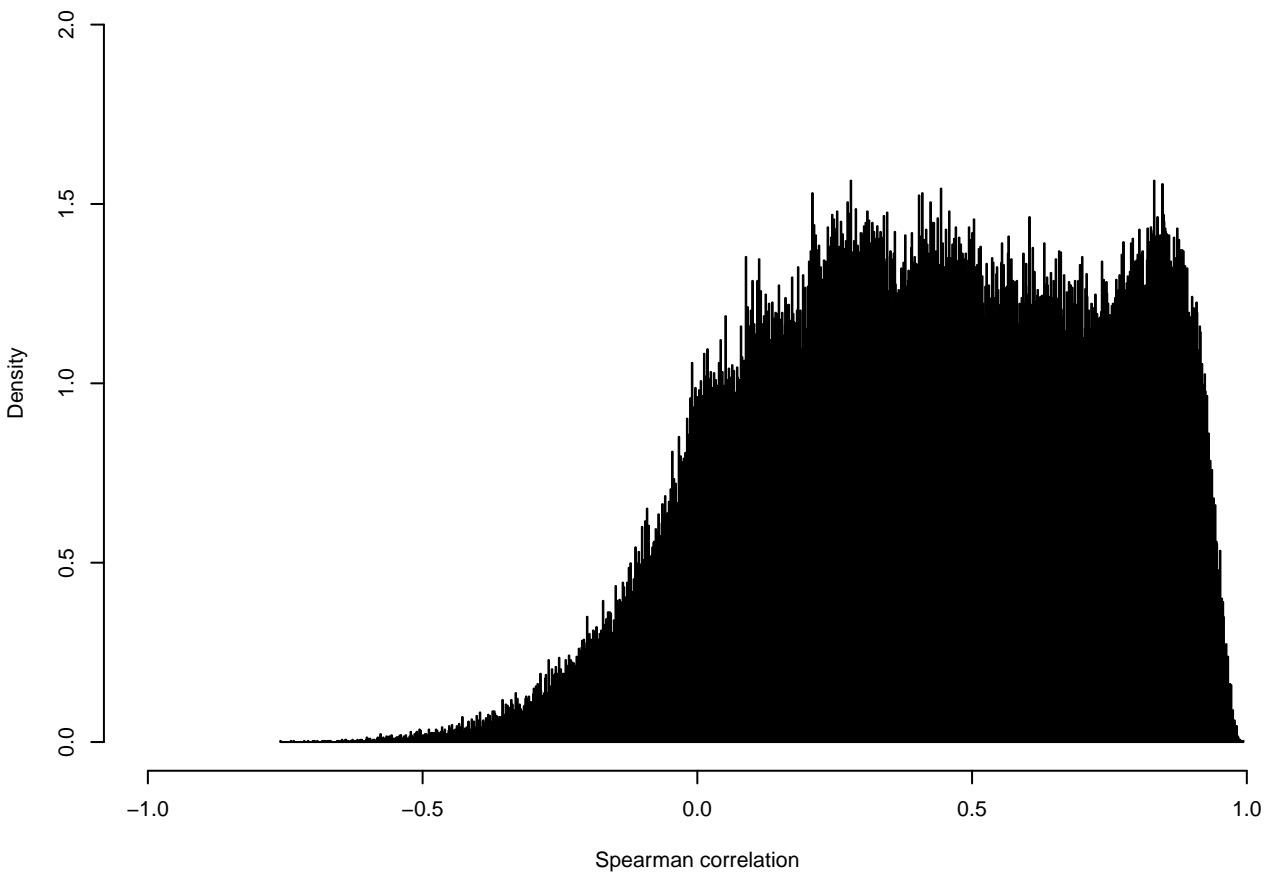

Supplement: Supplementary file 1 — Figure S1. Histograms of Spearman correlations between normalization factors and raw counts of non-differentially abundant genes (non-DAGs). Spearman correlations were compute per gene in the Human gut I, for group size 10+10, with 10% of effects divided equally between the two group, and fold-change 3. Affected genes were randomly selected in 100 iterations. The following methods are included in the figure trimmed mean of M-values (TMM), relative log expression (RLE), cumulative sum scaling (CSS), reversed cumulative sum scaling (RCSS), upper quartile (UQ), median (Med) and total count (TC). (PDF 76 kb) [file 12864_2018_4637_MOESM1_ESM.pdf]

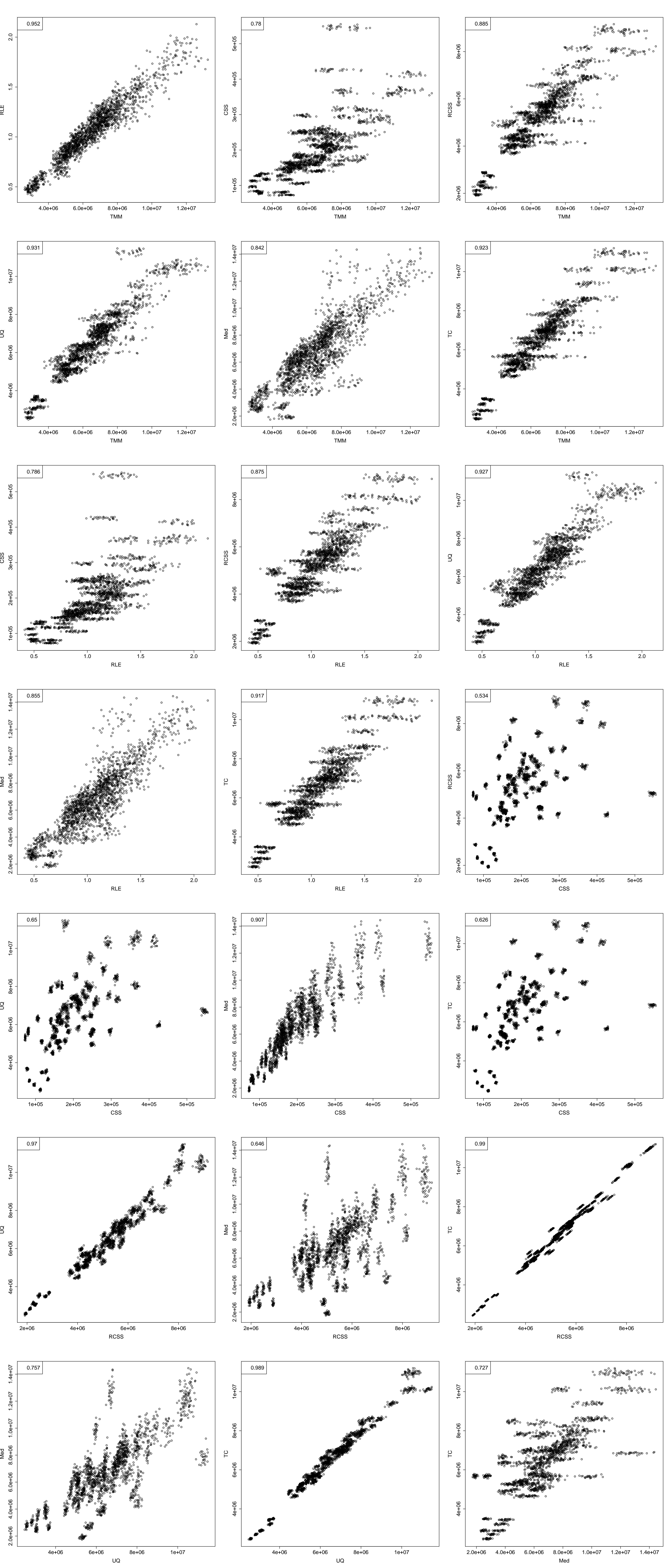

Supplement: Supplementary file 2 — Figure S2. Scatterplot of normalization factors for each pair of scaling methods. Normalization factors estimated per sample in the Human gut I, for group size 10+10, with 10% of effects divided equally between the two group, and fold-change 3. Affected genes were randomly selected in 100 iterations. The number on the top-left of each plot indicates the Spearman correlation for the normalization factors presented in the plot. The following methods are included in the figure trimmed mean of M-values (TMM), relative log expression (RLE), cumulative sum scaling (CSS), reversed cumulative sum scaling (RCSS), upper quartile (UQ), median (Med) and total count (TC). (PDF 316 kb) [file 12864_2018_4637_MOESM2_ESM.pdf]

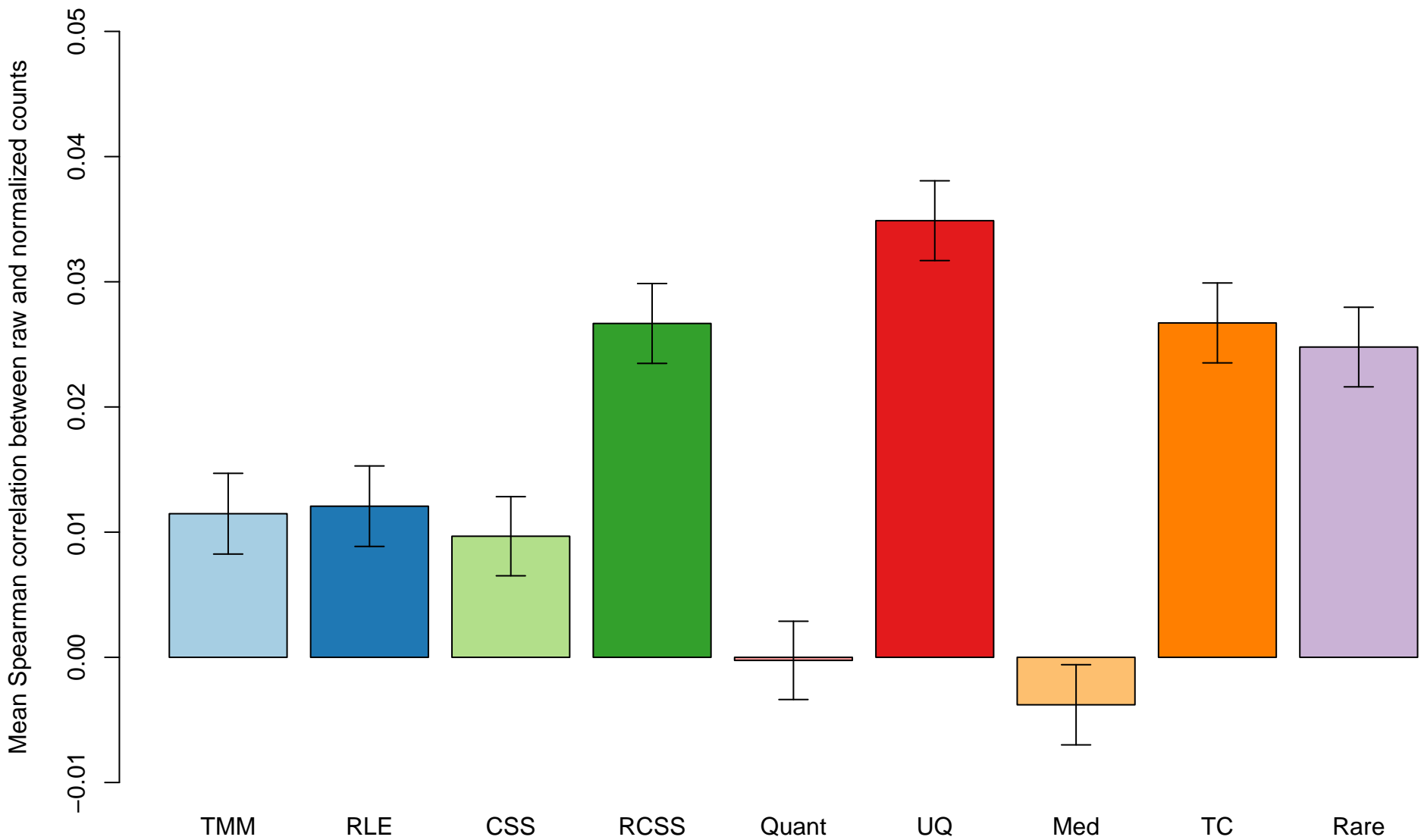

Supplement: Supplementary file 3 — Figure S3. Mean Spearman correlation between raw and normalized counts. Spearman correlations were compute per gene before and after normalization in the Human gut I, for group size 10+10, with 10% of effects divided equally between the two group, and fold-change 3. Affected genes were randomly selected in 100 iterations. The following methods are included in the figure trimmed mean of M-values (TMM), relative log expression (RLE), cumulative sum scaling (CSS), reversed cumulative sum scaling (RCSS), quantile-quantile (Quant), upper quartile (UQ), median (Med), total count (TC) and rarefying (Rare). (PDF 8 kb) [file 12864_2018_4637_MOESM3_ESM.pdf]

Effect Size for DAGs

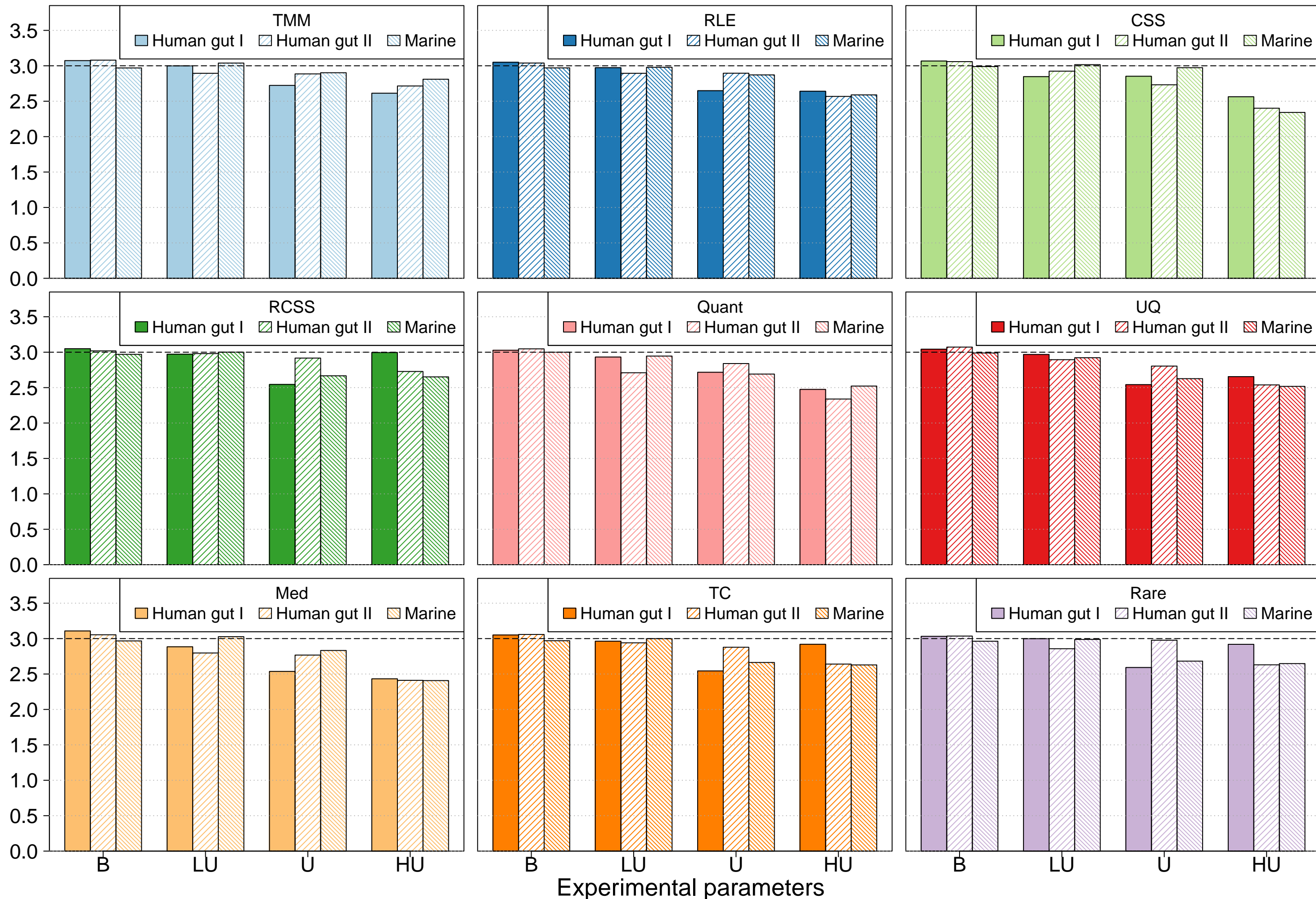

Supplement: Supplementary file 6 — Figure S4. Effect size analysis of DAGs. Estimated effect size of differentially abundant genes (DAGs) (y-axis) for different distribution of effects between groups (x-axis): balanced (‘B’) with 10% of effects divided equally between the two groups, lightly-unbalanced (’LU’) with effects added 75%-25% in each group, unbalanced (‘U’) with all effects added to only one group, and heavily-unbalanced (’HU’) with 20% of effects added to only one group (x-axis). The results were based on resampled data consisting of two groups with 10 samples in each, and an average fold-change of 3. Three metagenomic datasets were used Human gut I, Human gut II and Marine. The following methods are included in the figure trimmed mean of M-values (TMM), relative log expression (RLE), cumulative sum scaling (CSS), reversed cumulative sum scaling (RCSS), quantile-quantile (Quant), upper quartile (UQ), median (Med), total count (TC) and rarefying (Rare). (PDF 436 kb) [file 12864_2018_4637_MOESM6_ESM.pdf]

tFDR at fix eFDR (p-values adjusted using Benjamini–Yekutieli correction)

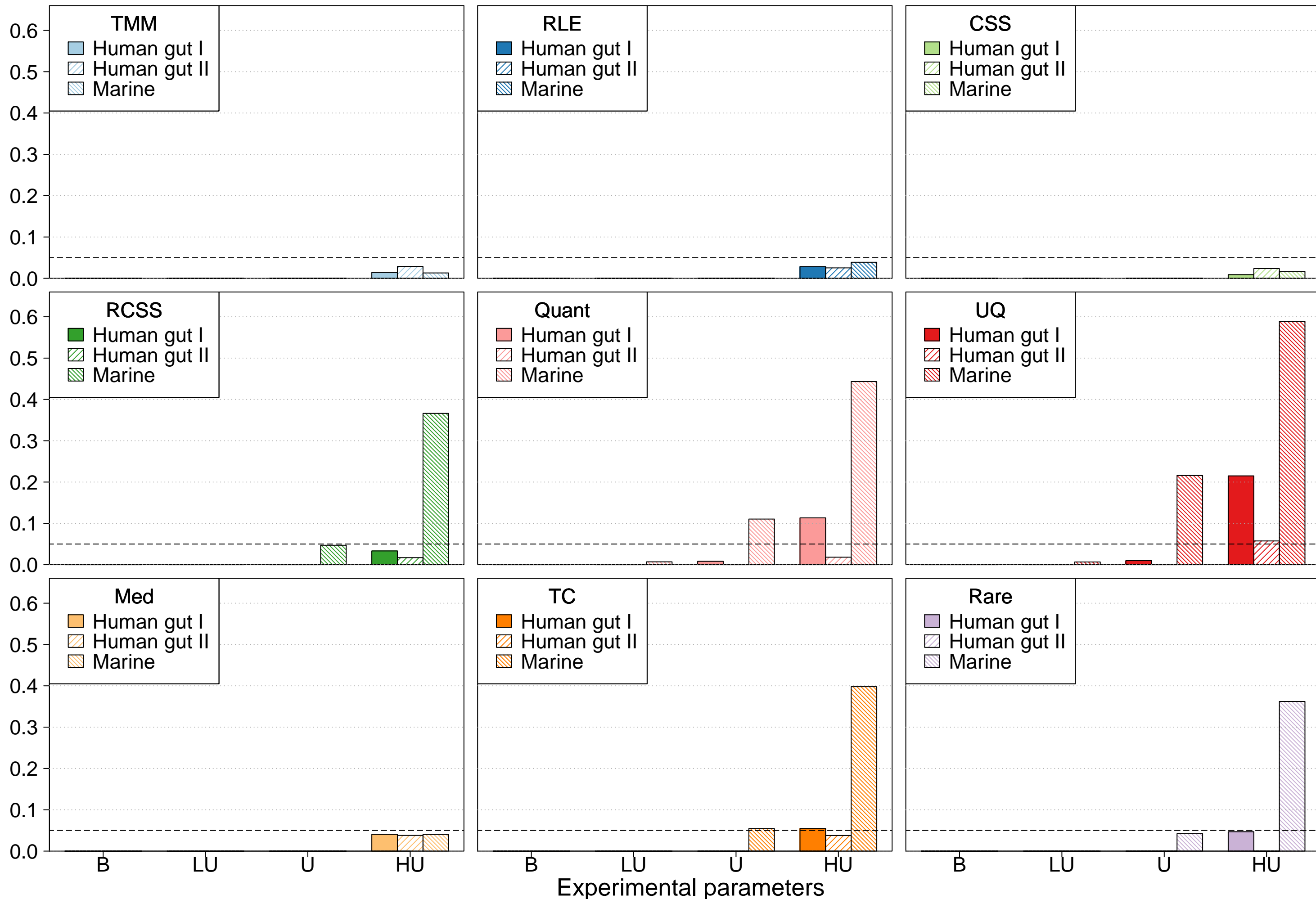

Supplement: Supplementary file 8 — Figure S5. True false discovery rate for p-values adjusted using Benjamini-Yekutieli method at an estimated false discovery rate of 0.05 (y-axis) for different distribution of effects between groups (x-axis): balanced (‘B’) with 10% of effects divided equally between the two groups, lightly-unbalanced (’LU’) with effects added 75%-25% in each group, unbalanced (‘U’) with all effects added to only one group, and heavily-unbalanced (’HU’) with 20% of effects added to only one group. The results were based on resampled data consisting of two groups with 10 samples in each, and an average fold-change of 3. Three metagenomic datasets were used Human gut I, Human gut II and Marine. The following methods are included in the figure trimmed mean of M-values (TMM), relative log expression (RLE), cumulative sum scaling (CSS), reversed cumulative sum scaling (RCSS), quantile-quantile (Quant), upper quartile (UQ), median (Med), total count (TC) and rarefying (Rare). (PDF 40 kb) [file 12864_2018_4637_MOESM8_ESM.pdf]

tFDR at fix eFDR (p-values adjusted using Storey q-values)

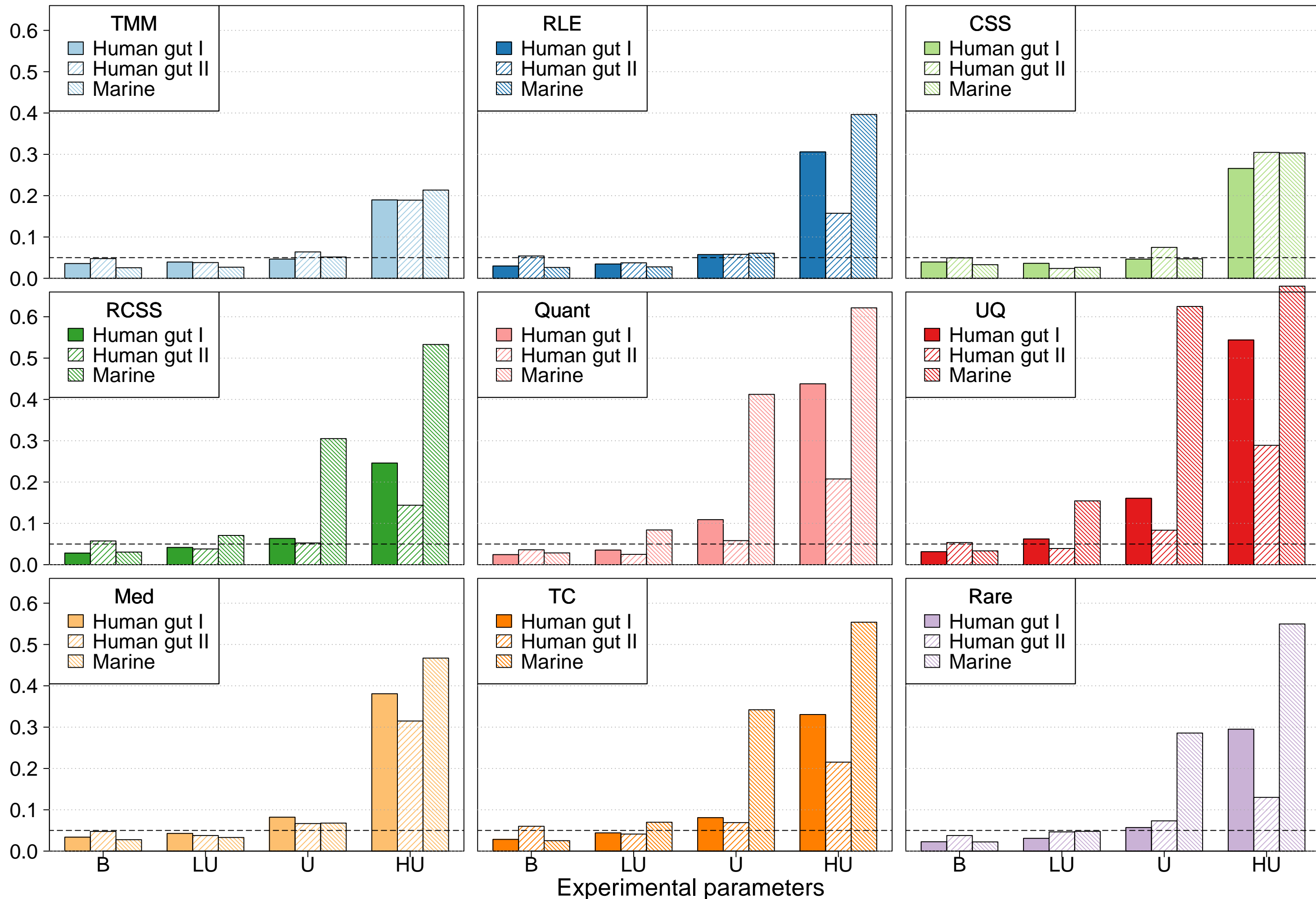

Supplement: Supplementary file 9 — Figure S6. True false discovery rate for p-values adjusted using Storey q-values method at an estimated false discovery rate of 0.05 (y-axis) for different distribution of effects between groups (x-axis): balanced (‘B’) with 10% of effects divided equally between the two groups, lightly-unbalanced (‘LU’) with effects added 75–25% in each group, unbalanced (‘U’) with all effects added to only one group, and heavily-unbalanced (‘HU’) with 20% of effects added to only one group. The results were based on resampled data consisting of two groups with 10 samples in each, and an average fold-change of 3. Three metagenomic datasets were used Human gut I, Human gut II and Marine. The following methods are included in the figure trimmed mean of M-values (TMM), relative log expression (RLE), cumulative sum scaling (CSS), reversed cumulative sum scaling (RCSS), quantile-quantile (Quant), upper quartile (UQ), median (Med), total count (TC) and rarefying (Rare). (PDF 132 kb) [file 12864_2018_4637_MOESM9_ESM.pdf]
