# Supplementary material for: Comparison of normalization methods for the analysis of metagenomic gene abundance data
Source: BMC Genomics. 2018 Apr 20;19:274. doi: 10.1186/s12864-018-4637-6 (PMC5910605; doi:10.1186/s12864-018-4637-6)
Supplement: Supplementary file 4 — Table S1. True positive rate at a fixed false positive rate of 0.01 for a group size of 10+10. (PDF 16 kb) [file 12864_2018_4637_MOESM4_ESM.pdf]

Table S1 True positive rate at a fixed false positive rate of 0.01 for a group size of $10 + 10$.

**fold change: 3**	Human gut I				Human gut II				Marine			
Method	B	LU	U	HU	B	LU	U	HU	B	LU	U	HU
TMM	0.62	0.61	0.58	0.48	0.63	0.66	0.59	0.43	0.66	0.66	0.61	0.54
RLE	0.63	0.60	0.55	0.42	0.62	0.64	0.60	0.46	0.66	0.65	0.59	0.49
CSS	0.61	0.59	0.53	0.39	0.65	0.66	0.55	0.36	0.69	0.67	0.61	0.49
RCSS	0.61	0.58	0.56	0.43	0.62	0.66	0.59	0.47	0.65	0.61	0.50	0.33
quantile-quantile	0.69	0.65	0.56	0.34	0.64	0.63	0.54	0.38	0.67	0.62	0.46	0.27
upper quartile	0.61	0.57	0.49	0.28	0.63	0.66	0.56	0.37	0.66	0.58	0.42	0.25
median	0.62	0.58	0.50	0.33	0.64	0.65	0.52	0.29	0.68	0.65	0.59	0.43
total count	0.61	0.58	0.54	0.39	0.63	0.65	0.57	0.42	0.65	0.61	0.49	0.33
rarefying	0.63	0.60	0.57	0.42	0.49	0.49	0.44	0.34	0.66	0.61	0.50	0.34
**fold change: 5**	Human gut I				Human gut II				Marine			
Method	B	LU	U	HU	B	LU	U	HU	B	LU	U	HU
TMM	0.72	0.72	0.70	0.60	0.83	0.81	0.79	0.65	0.78	0.77	0.75	0.64
RLE	0.72	0.72	0.67	0.54	0.83	0.79	0.79	0.68	0.78	0.77	0.73	0.57
CSS	0.74	0.72	0.63	0.46	0.84	0.80	0.71	0.42	0.81	0.80	0.69	0.51
RCSS	0.70	0.70	0.66	0.53	0.82	0.81	0.77	0.65	0.77	0.70	0.59	0.37
quantile-quantile	0.78	0.75	0.65	0.41	0.79	0.77	0.67	0.47	0.80	0.71	0.52	0.29
upper quartile	0.71	0.68	0.59	0.36	0.82	0.79	0.73	0.50	0.78	0.63	0.45	0.24
median	0.72	0.68	0.57	0.33	0.83	0.79	0.68	0.38	0.80	0.78	0.67	0.46
total count	0.71	0.70	0.64	0.48	0.82	0.80	0.76	0.56	0.77	0.70	0.57	0.36
rarefying	0.72	0.72	0.66	0.51	0.69	0.66	0.63	0.50	0.77	0.70	0.58	0.37

B: balanced, 10% effects added. 50% in each group,
LU: lightly-unbalanced, 10% effects added. 75%-25% is each group,
U: unbalanced, 10% effects added. 100% in only one group,
HU: heavily-unbalanced, 20% effects added. 100% in only one group.
