# Supplementary material for: Comparison of normalization methods for the analysis of metagenomic gene abundance data
Source: BMC Genomics. 2018 Apr 20;19:274. doi: 10.1186/s12864-018-4637-6 (PMC5910605; doi:10.1186/s12864-018-4637-6)
Supplement: Supplementary file 5 — Table S2. False positive rate at a fix true positive rate of 0.50 for a group size of 10+10. (PDF 16 kb) [file 12864_2018_4637_MOESM5_ESM.pdf]

Table S2 False positive rate at a fix true positive rate of 0.50 for a group size of $10 + 10$.

**fold change: 3**	Human gut I				Human gut II				Marine			
Method	B	LU	U	HU	B	LU	U	HU	B	LU	U	HU
TMM	9.5E-4	1.3E-3	2.5E-3	1.4E-2	2.2E-3	1.1E-3	3.3E-3	1.6E-2	0	3.2E-4	6.3E-4	3.6E-3
RLE	9.5E-4	1.4E-3	4.1E-3	2.8E-2	2.2E-3	1.1E-3	3.3E-3	1.6E-2	0	3.2E-4	9.5E-4	1.2E-2
CSS	1.9E-3	2.9E-3	7.1E-3	3.7E-2	1.7E-3	1.1E-3	5.6E-3	4.1E-2	3.2E-4	3.2E-4	1.3E-3	1.1E-2
RCSS	1.3E-3	2.4E-3	3.5E-3	2.6E-2	2.2E-3	2.2E-3	3.3E-3	1.4E-2	3.2E-4	6.3E-4	1.1E-2	8.6E-2
quantile-quantile	3.2E-4	9.5E-4	4.6E-3	5.2E-2	2.2E-3	2.2E-3	7.8E-3	3.6E-2	3.2E-4	1.3E-3	2.0E-2	1.3E-1
upper quartile	1.3E-3	2.2E-3	1.2E-2	1.0E-1	2.2E-3	2.2E-3	5.6E-3	3.6E-2	3.2E-4	1.3E-3	5.0E-2	2.0E-1
median	1.6E-3	3.2E-3	1.0E-2	6.6E-2	2.2E-3	2.2E-3	8.9E-3	6.7E-2	3.2E-4	3.2E-4	2.2E-3	3.1E-2
total count	1.1E-3	1.9E-3	4.8E-3	3.9E-2	2.2E-3	1.1E-3	4.4E-3	2.3E-2	3.2E-4	6.3E-4	1.2E-2	9.8E-2
rarefying	9.5E-4	1.3E-3	3.0E-3	2.8E-2	1.1E-2	1.1E-2	1.6E-2	3.8E-2	3.2E-4	6.3E-4	9.8E-3	8.5E-2
**fold change: 5**	Human gut I				Human gut II				Marine			
Method	B	LU	U	HU	B	LU	U	HU	B	LU	U	HU
TMM	0	0	0	1.4E-3	0	0	0	2.5E-3	0	0	0	3.6E-4
RLE	0	0	1.6E-4	5.7E-3	0	0	0	1.3E-3	0	0	0	1.3E-3
CSS	0	1.6E-4	1.3E-3	1.9E-2	0	0	1.1E-3	2.3E-2	0	0	0	7.1E-3
RCSS	0	0	3.2E-4	6.1E-3	0	0	0	1.3E-3	0	0	1.9E-3	5.8E-2
quantile-quantile	0	0	9.5E-4	2.9E-2	0	0	1.1E-3	1.5E-2	0	3.2E-4	7.5E-3	9.9E-2
upper quartile	0	0	1.9E-3	6.1E-2	0	0	0	1.0E-2	0	3.2E-4	2.9E-2	2.2E-1
median	0	3.2E-4	3.0E-3	6.9E-2	0	0	1.1E-3	2.9E-2	0	0	3.2E-4	2.5E-2
total count	0	0	3.2E-4	1.4E-2	0	0	0	5.0E-3	0	0	2.4E-3	7.5E-2
rarefying	0	0	3.2E-4	8.4E-3	1.1E-3	1.1E-3	2.8E-3	1.0E-2	0	0	1.9E-3	6.3E-2

B: balanced, 10% effects added. 50% in each group,
LU: lightly-unbalanced, 10% effects added. 75%-25% is each group,
U: unbalanced, 10% effects added. 100% in only one group,
HU: heavily-unbalanced, 20% effects added. 100% in only one group.
