# Supplementary material for: Comparison of normalization methods for the analysis of metagenomic gene abundance data
Source: BMC Genomics. 2018 Apr 20;19:274. doi: 10.1186/s12864-018-4637-6 (PMC5910605; doi:10.1186/s12864-018-4637-6)
Supplement: Supplementary file 7 — Table S3. True false discovery rate at an estimated false discovery rate of 0.05 for a group size of 10+10. (PDF 16 kb) [file 12864_2018_4637_MOESM7_ESM.pdf]

Table S3 True false discovery rate at an estimated false discovery rate of 0.05 for a group size of $10 + 10$.

**fold change: 3**	Human gut I				Human gut II				Marine			
Method	B	LU	U	HU	B	LU	U	HU	B	LU	U	HU
TMM	0.022	0.025	0.032	0.109	0.038	0.033	0.040	0.123	0.019	0.019	0.032	0.131
RLE	0.021	0.027	0.039	0.190	0.042	0.034	0.044	0.118	0.020	0.021	0.037	0.258
CSS	0.027	0.025	0.029	0.119	0.036	0.022	0.050	0.193	0.019	0.020	0.026	0.156
RCSS	0.022	0.031	0.041	0.163	0.040	0.032	0.045	0.111	0.021	0.042	0.243	0.490
quantile-quantile	0.016	0.028	0.071	0.325	0.024	0.021	0.045	0.138	0.024	0.061	0.334	0.567
upper quartile	0.021	0.036	0.109	0.432	0.046	0.036	0.055	0.205	0.023	0.096	0.527	0.644
median	0.022	0.027	0.041	0.229	0.033	0.031	0.054	0.213	0.017	0.020	0.034	0.264
total count	0.021	0.032	0.049	0.240	0.038	0.037	0.055	0.167	0.020	0.042	0.262	0.514
rarefying	0.018	0.022	0.042	0.212	0.029	0.046	0.065	0.098	0.014	0.039	0.226	0.500
**fold change: 5**	Human gut I				Human gut II				Marine			
Method	B	LU	U	HU	B	LU	U	HU	B	LU	U	HU
TMM	0.027	0.027	0.040	0.103	0.040	0.041	0.048	0.138	0.019	0.016	0.033	0.158
RLE	0.025	0.029	0.059	0.220	0.039	0.032	0.048	0.088	0.018	0.018	0.048	0.305
CSS	0.033	0.047	0.071	0.338	0.039	0.048	0.093	0.340	0.018	0.021	0.077	0.460
RCSS	0.027	0.029	0.055	0.221	0.043	0.043	0.051	0.115	0.021	0.082	0.330	0.498
quantile-quantile	0.023	0.042	0.141	0.426	0.028	0.034	0.063	0.193	0.029	0.103	0.460	0.607
upper quartile	0.030	0.045	0.187	0.505	0.041	0.049	0.077	0.257	0.020	0.199	0.675	0.678
median	0.028	0.056	0.152	0.525	0.028	0.048	0.078	0.372	0.015	0.023	0.124	0.625
total count	0.027	0.034	0.093	0.307	0.042	0.046	0.070	0.193	0.020	0.093	0.382	0.532
rarefying	0.018	0.026	0.063	0.282	0.050	0.050	0.057	0.093	0.020	0.072	0.334	0.515

B: balanced, 10% effects added. 50% in each group,
LU: lightly-unbalanced, 10% effects added. 75%-25% is each group,
U: unbalanced, 10% effects added. 100% in only one group,
HU: heavily-unbalanced, 20% effects added. 100% in only one group.
